# BEATVIC, a body-oriented resilience therapy for individuals with psychosis: Short term results of a multi-center RCT

**DOI:** 10.1371/journal.pone.0279185

**Published:** 2022-12-21

**Authors:** Elisabeth C. D. van der Stouwe, Bertine de Vries, Laura A. Steenhuis, Clement O. Waarheid, Remon Jans, Steven de Jong, André Aleman, Gerdina H. M. Pijnenborg, Jooske T. Van Busschbach

**Affiliations:** 1 Rob Giel Onderzoekcentrum, University Center of Psychiatry, University Medical Center Groningen, University of Groningen, Groningen, The Netherlands; 2 Department of Neuroscience, University Medical Center Groningen, Cognitive Neuroscience Center, University of Groningen, Groningen, The Netherlands; 3 Department of Clinical Neuropsychology, Faculty of Behavioural and Social Sciences, University of Groningen, Groningen, The Netherlands; 4 Department of Psychotic Disorders, GGZ Drenthe, Assen, The Netherlands; 5 Lentis Psychiatric Institute, Lentis Research, Groningen, The Netherlands; 6 Department of Movement and Education, Windesheim University of Applied Sciences, Zwolle, The Netherlands; Prince Sattam Bin Abdulaziz University, College of Applied Medical Sciences, SAUDI ARABIA

## Abstract

**Background:**

Individuals with a psychotic disorder are at an increased risk of victimization, but evidenced-based interventions are lacking.

**Aims:**

A body-oriented resilience therapy (‘BEATVIC’) aimed at preventing victimization was developed and its effectiveness was assessed in a multicenter randomized controlled trial.

**Methods:**

105 people with a psychotic disorder were recruited from six mental health centers. Participants were randomly allocated to 20 BEATVIC group sessions (n = 53) or befriending group sessions (n = 52). Short term effects on risk factors for victimization (e.g. social cognitive deficits, inadequate interpersonal behavior, low self-esteem, internalized stigma, aggression regulation problems), physical fitness and secondary outcomes were expected. At six-month follow-up, the effect on victimization (either a 50% reduction or an absence of victimization incidents) was examined.

**Results:**

Intervention-dropout was 28.30% for BEATVIC and 39.62% for befriending. In both conditions the majority of participants (60.5% BEATVIC vs 62.9% befriending) showed a reduction or absence of victimization incidents at six months follow-up, which was not significantly different according to condition. Multilevel analyses revealed no main effect of time and no significant time x group interaction on other outcome measures. Per protocol analyses (participants attending ≥ 75% of the sessions) did not change these results.

**Conclusions:**

Although a reduction or absence of victimization was found at short term follow-up for the majority of participants, BEATVIC was not more effective than the active control condition. No short-term additional effects on risk factors of victimization were found. Analysis of the data at 2-year follow-up is warranted to investigate possible effects in the long-term.

**Trial registration number:**

Current Controlled Trials: ISRCTN21423535.

## Introduction

Individuals with a psychotic disorder are more susceptible to falling victim to a crime (‘victimization’) than people from the general population [1]. The five year prevalence rate for people with a psychotic disorder is 22% for violent victimization, 32% for sexual victimization and 20% for non-violent crime: this is four to six times as high as in the general population [2]. Victimization in people with psychosis is associated with substance abuse, depression [3], severe symptomatology and poorer prognosis [4]. An intervention focused on prevention of victimization is vital for this population.

To date, no evidence-based intervention aimed at preventing victimization in individuals with psychosis is available. Victimization is a form of trauma and may lead to PTSD symptoms, potentially causing a large burden on mental health [5]. PTSD symptoms have been associated with a worse prognosis and impaired daily functioning in people with psychosis [6]. Although there are effective evidence based trauma-focused therapies for this group [7] that lead to a reduction of revictimization [8], prevention of any victimization is preferred. For this aim BEATVIC was developed: a body-oriented resilience therapy with kickboxing exercises in group format. The therapy uses a body-oriented approach [9–11] combining elements of physical exercise, assertiveness training and social cognition training, allowing participants to recognize their own emotional and behavioral reactions in social situations, and to practice with new and adequate social behavior in a safe environment.

BEATVIC aims to prevent victimization and is based on a model that addresses risk factors for victimization which may be modifiable and feasible to improve [12, 13]. The first risk factor is social cognitive impairment, as individuals with a psychotic disorder show a deficiency in recognizing facial expressions, body language, mentalizing and theory of mind (ToM). This deficit could prevent accurate judgement of a risky social situation and/or leads to conflicts that may result in victimization [14, 15]. Another factor is self-stigma, which is associated with low self-efficacy [16], low self-esteem and reduced empowerment [17]. These factors may lead to difficulties in standing up for oneself in social situations and increasing the chance of victimization [18]. Furthermore, sometimes perpetration and victimization go hand in hand [19]. In 24% of the violent crime cases the victims were also perpetrators [20, 21]. Some individuals with a psychotic disorder may exhibit aggression regulation problems, or reduced clinical insight, which can be associated with aggressive behavior [22] and therefore enhancing risk for victimization [23]. To summarize these risk factors we constructed a model (see [12] on which the BEATVIC intervention was based).

In the feasibility study of BEATVIC, the intervention was deemed acceptable and feasible [13]. Participants attended an average of 85% sessions and evaluated BEATVIC as helpful in gaining self-esteem and assertiveness. Based on this study, small adjustments were made to the intervention and the research protocol. The current paper presents the results of a randomized controlled trial investigating the short term effectiveness of BEATVIC [12] comparing pre-, post- and the first follow-up assessment at six months. Short term effects on risk factors for victimization (e.g. social cognition, interpersonal behavior, self-esteem, internalized stigma, insight and aggression regulation) were expected, as these are directly targeted. Because incidents of violence do not occur often, it was hypothesized that effects on victimization would become apparent at six-month follow-up. A further decrease after one and two years (not here reported), is also expected. In addition, we examined effects on secondary outcome variables (e.g. recovery, social participation, quality of life, symptoms and PTSS symptoms), and on physical outcomes (e.g. physical activity and fitness).

## Materials and methods

The study is funded by the Netherlands Organization for Scientific Research (NWO grant nr 432-12-807). All procedures involving patients were approved by the local ethical committee (University Medical Center of Groningen, The Netherlands; METc protocol number: NL52202.042.15). The authors assert that all procedures contributing to this work comply with the ethical standards of the relevant national and institutional committees on human experimentation and with the Helsinki Declaration of 1975, as revised in 2008. Although the trial was registered prospectively (Current Controlled Trials: ISRCTN21423535), it was classed as retrospective due to a delay in finalizing the registration in time for the recruitment to start. However, no deviations from the protocol were made as presented in the trial register. Recruitment of participants for baseline, post-treatment and follow-up assessments, lasted from February 2016 until March 2021. Recruitment stopped when the minimum amount of participants were included in both conditions and no further participants were found to be included in the study. For further details of this study, see the protocol paper [12].

### Design

The study is designed as a multi-center randomized controlled trial including a pretest (T0), a posttest (T1) and three follow up assessments respectively at 6 (T2), 18 (T3) and 30 (T4) months. The current article presents the findings of assessment points T0, T1 and T2. Patients allocated to the intervention group receive the BEATVIC group training and patients allocated to the control group receive befriending sessions.

### Participants and setting

Participants were recruited from six mental health care facilities in the Netherlands. The following inclusion criteria were used to identify potential participants:

- A diagnosis in the psychotic spectrum, according to DSM-IV-TR criteria. This was verified by use of the mini-SCAN.
- At least 18 years of age.
- Having the ability to provide written informed consent.

In addition, the following exclusion criteria were used to exclude participants:

- Severe psychotic symptoms (mean positive symptoms > 5 measured by PANSS)
- Substance dependence (not substance abuse), also verified by use of the mini-SCAN.
- Co-morbid neurological disorder of personality disorder, as verified by onsite therapist.
- Estimated IQ < 70, as judged by onsite therapist.
- Pregnancy.

The sample size was computed a-priori using the IBM SPSS Sample Power program (http://www.power-analysis.com/about_biostat.htm). The effect size was set at 0.5, because a lower effect size would not be considered as clinically relevant. In order to find a medium effect size on victimization with an alpha of 0.05 and a power of 0.80, a minimum of 48 participants per condition was required. Considering a drop-out of 25%, it was aimed to include a total of 120 participants in the current trial.

### Intervention

#### BEATVIC

In short, BEATVIC consists of five modules covering 20 weekly group sessions of 75 minutes led by a therapist trained in body and movement-oriented interventions (i.c. a psychomotor therapist (www.psychomot.org/) and an expert by experience). Sessions include a warming-up, technical kickboxing exercises and thematic exercises, a cooling-down and a group discussion to help transfer the acquired skills to application in daily life.

In the first module, basic kickboxing techniques are taught and discussion themes are centered around body posture, self-stigma and setting boundaries. The second module, ‘Recognizing dangerous behavior’, aims to improve social cognition and insight. The third module, ‘How others see me’, emphasizes patients’ own behavior and how they may appear to others. In the fourth module ‘Coping with aggression’ patients learn to regulate their own aggression and how to deal with aggressive behavior of others. In the final module, exercises were tailored to the specific needs of the group.

#### Control condition

The control group participated in 20 weekly ‘befriending’ sessions of 75 minutes consisting of five modules: ‘Introduction’, ‘Media’, ‘Hobbies’, ‘Lifestyle’ and ‘Repetition and follow-up’. Goal was to provide a welcoming environment in which patients can socially interact. Befriending is deemed a credible and acceptable control condition with regard to enjoyment of therapy, expectancy and drop-out rate [24].

### Primary outcomes

#### Victimization

Treatment response (coded as yes or no) was assessed with the revised Conflict Tactics Scale (CTS2) [25], which assesses different forms of victimization and perpetration in the preceding year. The original CTS2 assesses partner violence but in this study an adapted version was used also assessing other social interactions. For this study, perpetration was not examined. The CTS2 assesses victimization in the following subdomains: psychological aggression, physical assault, sexual coercion, physical injury, and negotiation. Negotiation was not considered part of victimization and not examined. Participants had to indicate the frequency of victimization on 39 items in the previous six months, ranging from never, once, twice, 3–5, 6–10, 11–20 and > 20 times. We calculated a frequency score for total victimization using a midpoints substation scoring method [26]. Using percentage change scores in frequency from T0 to T2, the treatment response (yes/no) was coded. Treatment response on the CTS2 was defined as having reported no victimization incidents at T2 or reporting a 50% reduction in victimization at T2 compared to T0 (in line with previous research and expert discussions [27]).

To compare victimization rates with the general population, the victimization subscale of the Safety Monitor (IVM), a victimization survey that is the Dutch equivalent of the International Crime Victims Survey (ICVS [28]) was administered at baseline. Participants were asked whether they experienced different types of victimization in the past 12 months and in the past five years.

#### Social cognition

The Faux Pas task is a Theory of Mind (ToM) test [29], in which participants have to indicate whether a faux pas is present in a story. If present, participants are asked questions to assess their understanding of this faux pas. In the analyses the percentage of correct answers was used.

#### Aggression regulation

The Self-expression and Control Scale (SEC) is a 40-item Dutch translation of 4 subscales (internalizing and externalizing anger, and control of internalizing and externalizing anger) of the State-Trait Anger Expression Inventory [30]. In the analyses the total score on each of four subscales was used.

#### Internalized stigma

Self-stigma was assessed with the Internalized Stigma of Mental Illness Scale (ISMI); [31]. The ISMI consists of the subscales stereotype endorsement, perceived discrimination, alienation, social withdrawal and stigma resistance and contains 29 items.

#### Social behavior

The Scale for Interpersonal Behavior (SIB [32]) measures social anxiety and social skills using 50 items. The total frequency of social anxiety, and total social discomfort score was used for the analyses.

#### Self-esteem

To assess self-esteem the 20-item Self-Esteem Rating Scale-Short Form (SERS-SF) was used [33]. Ten items asses positive self-esteem and ten items measure negative self-esteem.

#### Insight

The 8-item Psychosis Insight Scale (PI [34]) measures insight in people with a psychotic disorder on three dimensions: attribution of symptoms, need for treatment and awareness of illness.

### Secondary outcomes

Most secondary outcomes were administered at T0, T1 and T2, but physical activity and endurance only at T0 and T1.

#### Quality of life

The total score of the 12-item Manchester Short Assessment of Quality of Life (MANSA) was used [35], which was designed for people with severe mental illness.

#### Recovery

The total score of the Dutch 12-item National Recovery Scale (NRS [36]) was used (based on the Questionnaire about the Process of Recovery [37]).

#### Societal participation

The 78-item Social Functioning Scale (SFS); [38] measures social participation and social functioning. The raw total score of 75 of the 78 items was used.

#### Symptoms

The 13-item Brief Negative Symptom Scale (BNSS) [39] assesses negative symptoms using six subscales: anhedonia, distress, asociality, avolition, blunted affect and alogia. The mean score of subscales was used.

#### Trauma

The total score of the 10-item Trauma Screening Questionnaire (TSQ [40]), was used to assess post-traumatic stress symptoms for those confirming experiencing or witnessing a life-threatening or shocking event in their lifetime.

#### Physical activity

All participants were asked to wear a pedometer (Yamax EX 510 [41]) between the intake and the T0 assessment and between post-treatment and the T1 assessment. The mean of the three most active days was used (in line with previous research [42]).

#### Aerobic fitness

We used the 28 level Modified Shuttle Test (MST), based on the 20 level Shuttle Test [43] with eight additional lower levels to accommodate for people with lower aerobic capacity [44]. The outcome measure is the amount of meters a participant can walk or run between two points.

### Covariates

#### Demographic characteristics

At T0 the participants indicated their age, gender, living situation, age of onset, number of psychotic episodes, number of hospital admissions, amount of family contact, and sport participation.

#### Substance use

The total score of the 11-item Dutch Screening risk of substance dependence [45] was administered at T0, T1 and T2.

### Procedure

Treating clinicians will screen patients based on the in- and exclusion criteria. Patients who meet the criteria will be contacted and asked if they are interested in participating in the study. Information letters will be sent to patients who are interested. Subsequently, patients have a two-week period to consider final participation. After written informed consent was obtained, diagnosis and absence of substance dependence and severe psychotic symptoms were verified by the miniSCAN and PANSS respectively.

Directly after baseline, participants were randomized by an independent randomizer. This was completed separately for each treatment center, in order to assure a comparable number of participants in both groups. The randomization procedure took place once the number of patients required for two groups (experimental and control) was included (20 patients per centre) or when the first participant from that specific center was included more than six weeks ago whilst at least 12 participants from that center were included overall. An independent team of researchers not involved in the trial performed the randomization procedure, which was stratified by gender and participation in an fMRI substudy [46].

To gain insight in the short-term effects, assessments on victimization incidents were conducted at baseline (T0) and six months post-treatment (T2). All other primary outcomes were assessed at T0, T1 and T2. At all sites, trained interviewers were available who were blinded to the study condition. BEATVIC trainers and patients cannot be blinded after treatment allotment. Patients were instructed not to inform the assessor about the study condition they were allocated to.

### Statistical analyses

Demographic differences between groups were tested using Pearson chi-squared tests (categorical variables) and independent-samples T-tests (continuous variables). Continuous variables not normally distributed were tested using Mann-Whitney U tests. Tests were conducted two-tailed, with a significance level set at α = 0.05.

The primary outcome treatment response (yes/no) on total victimization was analyzed using two separate logistic regression analyses with treatment condition as independent variable. All participants were included regardless of attendance (intention to treat; ITT). Two additional models were fitted adjusted for predictors of treatment response for total victimization. Predictors (including age, gender, supported housing, family contact, diagnosis, age of onset, number of psychotic episodes, number of hospital admissions, participating in a sport, and group preference (kickboxing, befriending or no preference)) were entered in the logistic regression model using backward elimination.

The effects of BEATVIC on the continuous outcome measures were assessed with multilevel analyses as assessments (level 1) were nested within individuals (level 2) and clustered within sites (level 3). In MLwiN [47] a separate 3-level model was constructed for each of the outcome variables. The following predictors were entered as fixed effects: a) dummy variables representing time (T0, T1, T2); b) condition (BEATVIC, Befriending); and c) the interactions (T1*condition, T2*condition). The intercepts at levels 2 and 3, and the residual at level 1 were included as random effects. For each model it was tested whether the third level (site) was significant in the model by means of deviance tests [48]. If not, it was removed. To assess a main effect of time, significance testing was conducted using deviance tests between the model with time (T1 or T2), and without. Similarly, differences between BEATVIC and befriending at T1 and T2 were tested comparing deviance tests between the models *with* the interaction between time (T1 or T2) and condition (BEATVIC/befriending), and *without*. To correct for multiple testing, the significance level was set at α = 0.003 using Bonferroni (0.05/15 outcome measures).

Both multilevel and logistic regression analyses were repeated as per protocol, including participants who attended a minimum of 75% of the sessions (>15 sessions).

## Results

### Sample characteristics

**Fig 1:**
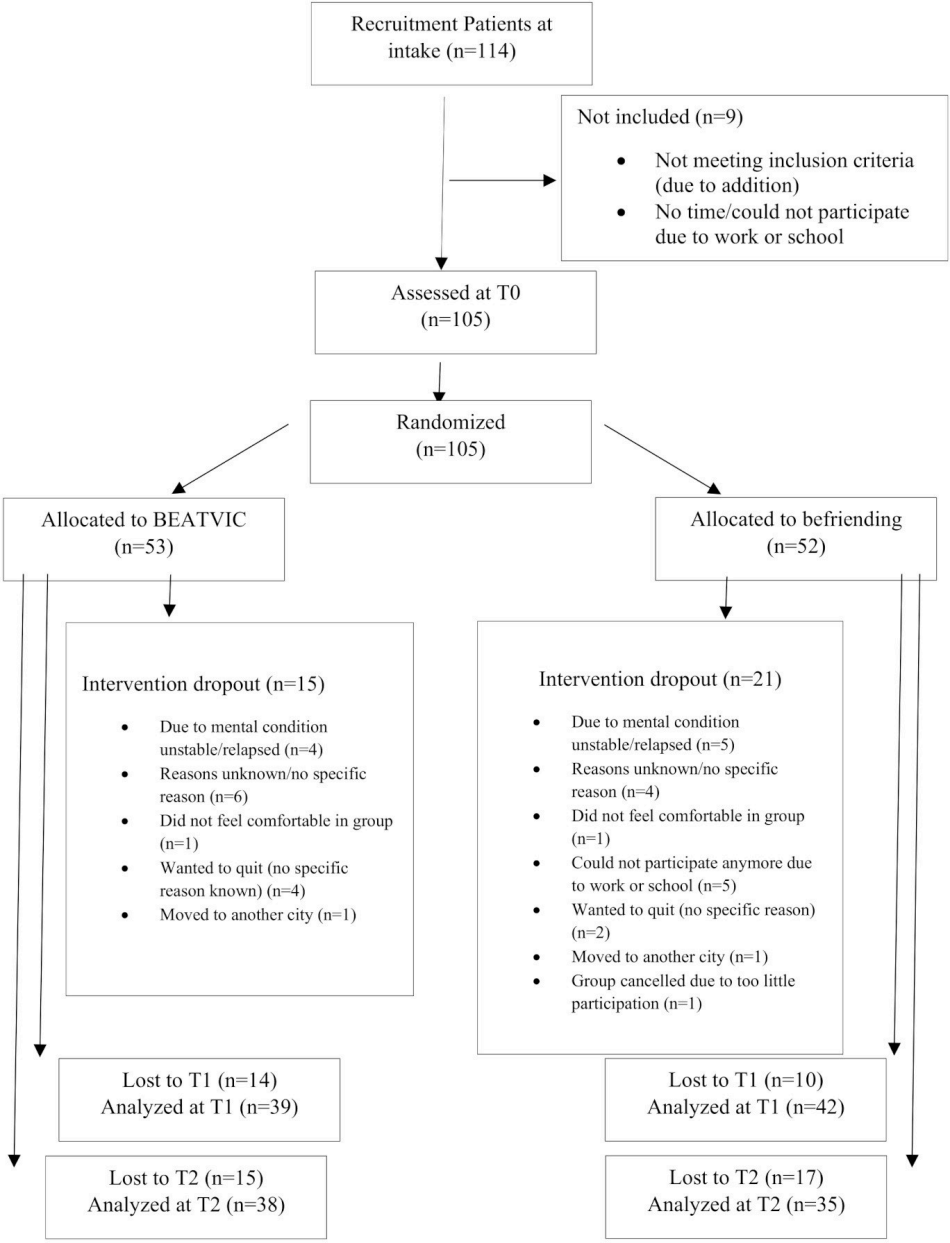
CONSORT diagram of participant flow.

In total, 105 participants were included of which 81 participants completed T1 and 73 completed the T2 assessment (Fig 1). Participants in BEATVIC and befriending did not differ significantly on baseline characteristics or victimization rates (IVM) (Table 1). There was no significant difference in the number of sessions attended (t = -1.89, df = 103, p = 0.06); BEATVIC mean 13.38 of 20 (SD 6.48), befriending 10.79 of 20 (SD 7.47). Attendance of 15 or more sessions was achieved by 60.38% of participants in BEATVIC and 40.38% of participants in befriending. Victimization incidents in both conditions consisted mostly of threats towards physical assault, followed by actual physical assault, with a small minority reporting sexual coercion.

**Table 1:** Demographic and clinical characteristics at baseline.

		N	Kickboxing % (n)	Befriending % (n)	*p*-value
N total		105	50.5 (53)	49.5 (52)	
Age mean (SD)		105	36.53 (11.27)	37.7 (12.02)	0.60
Gender	male	63	58.5 (31)	61.5 (32)	0.75
	female	42	41.5 (22)	38.5 (20)	
Supported housing	yes	28	28.8 (15)	25.5 (13)	0.70
	no	75	71.2 (37)	74.5 (38)	
Family contact	every week	83	84.9 (45)	73.1 (38)	0.21
	every month	14	7.5 (4)	19.2 (10)	
	every year or less	8	7.5 (4)	7.7 (4)	
Diagnosis	Schizophrenia	47	41.5 (22)	48.1 (25)	0.50
	Other psychotic disorder	58	58.5 (31)	51.9 (27)	
Age of onset	≤17	19	25.7 (9)	25.6(10)	0.56
	18–28	34	51.4 (18)	41.0 (16)	
	≥29	21	22.9 (8)	33.3(13)	
Number of psychotic episodes	1	18	18.8 (6)	31.6 (12)	0.18
	2	23	28.1 (9)	36.8 (14)	
	≥ 3	29	53.1 (17)	31.6 (12)	
Number of hospital admissions	1	14	25.8 (8)	25.0 (6)	0.99
	2–3	25	45.2 (14)	45.8 (11)	
	≥ 4	16	29.0 (9)	29.2 (7)	
Participating in a sport	yes	54	49.1 (26)	53.8 (28)	0.62
	no	51	50.9 (27)	46.2 (24)	
Group preference	kickboxing	43	43.4 (23)	38.5 (20)	0.40
	befriending	24	26.4 (14)	19.2 (10)	
	no preference	38	30.2 (16)	42.3 (22)	
IVM: Total Victimization (12 months)	0	91	88.7 (47)	84.6 (44)	0.54
	1	14	11.3 (6)	15.4 (8)	
IVM: Total Victimization (five years)	0	64	62.3 (33)	59.6 (31)	0.05
	1	26	20.8 (11)	28.8 (15)	
	2	9	5.7 (3)	11.5 (6)	
	3	6	11.3 (6)	0 (0)	
IVM: Victim of threats of violence (12 months)		105	4 (7.5)	3 (5.8)	0.72
IVM: Victim of threats of violence (five years)		105	14 (26.4)	11 (21.2)	0.53
IVM: Victim of physical assault (12 months)		105	2 (3.8)	2 (3.8)	0.99
IVM: Victim of physical assault (five years)		105	13 (24.5)	9 (17.3)	0.36
IVM: Victim of sexual harassment or assault (12 months)		105	0 (0)	3 (5.8)	0.07
IVM: Victim of sexual harassment or assault (five years)		105	8 (15.1)	7 (13.5)	0.81

Note.

IVM, victimization subscale of the Safety Monitor.

### Drop-out

For BEATVIC the attrition rate was 28.30% (15/53) and for befriending it was 39.62% (21/52). In both groups an equal number of participants dropped out due to their mental condition, unrelated to the intervention. As presented in the flow diagram (Fig 1) reasons for discontinuation of the therapy were unknown in respectively six and four cases because of study dropout.

Given the ITT design, participants were invited for post- and follow-up assessments irrespective of whether they had completed the intervention. Study drop-out on T1 and T2 was 24.5% and 30.2% in the BEATVIC condition compared with 21.2% and 32.7% in the befriending condition.

### Effect of site

No significant contribution of treatment site was found in both the regression models or in the multilevel models. As such, site was not added to the models, and removed as a third level.

### Logistic regression analyses

In Table 2, 60.5% participants from both BEATVIC and 62.9% of the befriending group reported a prevention of victimization, with no significant difference between groups (p = 0.84). In BEATVIC, 14 (60.9%) participants reported no victimization incidents at T2, and 9 (39.1%) participants reported a 50% reduction in incidents at T2. Adjusting for age, supported housing and family contact in the analyses did not change overall outcomes (p = 0.83). Rerunning the models for participants who completed at least 75% (>15) of sessions, did not change outcomes (p = 0.82). The model fit was tested on the model adjusted for covariates using the Hosmer and Lemeshow test, indicating a good fit (*X*^2^(8, 105) = 5.22, *p* = 0.73).

**Table 2:** Observed percentage of participants with treatment response at 6 month follow-up and results of the logistic regression analyses for primary outcome treatment response for total victimization.

		Beatvic		Befriending				95% C.I.	
		Responder N (%)	Non-responder N (%)	Responder N (%)	Non-responder N (%)	OR	p-value	Lower	Upper
Total victimization	ITT	23 (60.5)	15 (39.5)	22 (62.9)	13 (37.1)	0.91	0.84	0.35	2.33
	ITT-adjusted^1^	23 (60.5)	15 (39.5)	22 (62.9)	13 (37.1)	0.86	0.83	0.29	2.66
	PA	17 (60.7)	11 (39.3)	13 (65)	7 (35)	0.85	0.82	0.22	3.37

Note.

ITT, intention to treat analysis including all participants; ITT-adjusted, intention to treat analysis including all participants whilst correcting for predictors of treatment outcome; PA, per protocol analysis including only participants who participated in at least 75% of the sessions.

^1^, adjusted for age, supported housing & family contact.

### Multilevel analyses

Descriptive statistics for the risk factors and secondary outcomes at T0 and, if applicable, T1 and T2 are presented in Table 3. For the separate multilevel analyses per risk factor, no main effect of time was determined over the two periods (T0-T1 and T0-T2). Also no significant differences between groups over time (see Results in Table 4) were found.

**Table 3:** Means and standard deviations of continuous primary and secondary outcome measures.

	BEATVIC			Befriending		
	T0	T1	T2	T0	T1	T2
*Primary outcomes*						
N	53	39	38	52	42	35
FauxPas_%correct	74.11 (18.0)	79.53 (18.1)	75.98 (20.9)	78.32 (20.1)	80.81 (15.96)	84.63 (17.5)
SEC_IA	23.25 (7.6)	24.08 (7.8)	23.68 (8.3)	23.88 (6.8)	22.12 (5.53)	22.60 (6.9)
SEC_EA	16.70 (4.1)	16.85 (4.2)	16.70 (4.3)	17.50 (5.0)	16.21 (4.05)	16.29 (4.0)
SEC_CIA	29.91 (7.9)	29.36 (7.5)	28.41 (6.9)	28.65 (6.8)	30.95 (5.80)	29.37 (7.0)
SEC_CEA	31.51 (5.6)	31.21 (6.0)	30.92 (4.9)	31.3 (5.1)	32.57 (4.36)	31.51 (5.5)
ISMI_total	63.26 (14.5)	61.77 (16.5)	61.11 (15.3)	61.37 (13.0)	60.36 (12.82)	57.91 (12.6)
SIB_Tension	121.08 (39.7)	122.90 (40.1)	119.84 (40.5)	112.87 (34.1)	110.40 (34.04)	107.69 (31.5)
SIB_Frequency	130.00 (26.1)	140.26 (30.9)	135.89 (33.5)	134.75 (28.1)	133.19 (28.80)	131.63 (32.7)
SERS_negative	34.98 (13.3)	35.59 (13.1)	36.16 (12.8)	35.85 (13.6)	30.90 (11.71)	31.97 (12.1)
SERS_positive	46.91 (12.2)	47.54 (11.9)	49.08 (9.9)	45.56 (9.8)	47.36 (12.78)	47.89 (10.4)
PI_total	12.04 (3.1)	12.36 (3.5)	11.92 (3.5)	11.75 (3.4)	11.74 (3.36)	11.60 (2.9)
*Secondary outcomes*						
MANSA_total	56.38 (15.7)	54.77 (13.7)	57.27 (12.8)	56.69 (11.0)	60.57 (10.97)	54.94 (13.5)
NRS_total	92.02 (19.2)	93.08 (19.6)	93.62 (19.0)	91.35 (18.3)	98.60 (16.20)	95.09 (14.7)
SFS_total	125.53 (23.5)	124.67 (22.1)	126.32 (21.0)	125.62 (19.4)	130.52 (17.77)	131.94 (19.4)
BNSS_mean	1.11 (0.8)	1.16 (1.0)	1.44 (1.1)	1.11 (0.6)	1.37 (0.95)	1.14 (0.9)
TSQ_total	13.90 (3.0)	13.34 (3.0)	14.07 (3.2)	14.85 (2.6)	14.55 (2.66)	15.62 (2.2)
Pedometer	8059 (4097)	8123 (4709)	n.a.	9344 (4642)	7750 (4335)	n.a.
MST_level	10.06 (3.8)	10.11 (3.6)	n.a.	9.49 (3.7)	10.27 (4.38)	n.a.
MST_meters	975.8 (585.6)	926.9 (585.5)	n.a.	893.00 (607.2)	1031.6 (779.3)	n.a.

Note.

SEC_IA: Internalized anger subscale, SEC_EA: Externalized anger subscale, SEC_CIA: Control over internalized anger subscale, SEC_CEA: Control over externalized anger subscale, SIB_tension: Tension subscale, SIB_frequency: Frequency subscale, SERS_pos: Positive subscale, SERS_neg: Negative subscale, PI_total: Psychosis Insight Scale Total Score.

**Table 4:** Fixed and random effects (beta and standard error) on primary outcomes.

	Faux Pas	SEC_IA	SEC_EA	SEC_CIA	SEC_CEA	ISMI_sum	SIB_tension	SIB_frequency	SERS_pos	SERS_neg	PI_sum
Parameter	Beta (S.E.)										
Fixed effects											
Time factor											
Baseline (T0)	78.45 (2.60)	23.89 (0.98)	17.50 (0.60)	28.65 (0.98)	31.35 (0.72)	61.37 (1.93)	112.865 (5.00)	134.75 (4.05)	45.56 (1.55)	35.85 (1.76)	11.75 (0.46)
Post effect (T1)	0.93 (2.94)	-1.75 (0.92)	-1.22 (0.57)	2.07 (1.00)	1.02 (0.86)	-1.93 (1.67)	-5.74 (4.41)	-1.59 (4.53)	3.07 (1.50)	-5.91 (1.44)	-0.27 (0.41)
Post effect BEATVIC (T1)	3.55 (4.18)	2.06 (1.32)	1.55 (0.82)	-3.14 (1.43)	-1.55 (1.23)	-0.35 (2.40)	5.76 (6.32)	12.17 (6.48)	-2.59 (2.14)	6.92 (2.06)	0.30 (0.58)
Follow-up effect (T2)	3.14 (3.09)	-1.33 (0.98)	-0.69 (0.61)	0.78 (1.07)	-0.15 (0.91)	-3.68 (1.79)	-6.39 (4.72)	-3.23 (4.83)	3.20 (1.60)	-4.25 (1.54)	-0.21 (0.43)
Follow-up effect BEATVIC (T2)	-1.60 (4.27)	1.11 (1.37)	0.57 (0.86)	-2.60 (1.50)	-0.57 (1.28)	0.80 (2.50)	4.36 (6.61)	9.63 (6.76)	-1.30 (2.24)	5.11 (2.15)	-0.26 (0.61)
Random effects											
*Variances of*											
Level 2 –intercept	167.95 (35.32)	31.54 (5.57)	11.46 (2.05)	27.51 (5.25)	10.94 (2.59)	132.57 (22.31)	883.46 (149.78)	398.93 (84.82)	76.40 (13.77)	116.08 (19.03)	7.28 (1.24)
Level 1 –residual	18.36 (2.09)	7.15 (0.82)	22.00 (2.50)	16.35 (1.85)	179.03 (20.75)	48.89 (5.57)	44.63 (5.09)	3.58 (0.41)	60.66 (6.92)	422.60 (48.24)	454.03 (51.62)

Note.

SEC_IA: Internalized anger subscale, SEC_EA: Externalized anger subscale, SEC_CIA: Control over internalized anger subscale, SEC_CEA: Control over externalized anger subscale, SIB_tension: Tension subscale, SIB_frequency: Frequency subscale, SERS_pos: Positive subscale, SERS_neg: Negative subscale, PI_total: Psychosis Insight Scale Total Score. All effects were non-significant with *p*-values above 0.05.

### Continuous secondary outcomes

No significant main effect of time, and no differences between groups over time on the secondary outcome measures, were observed (see Results in Table 5).

**Table 5:** Fixed and random effects on secondary outcomes.

	MANSA	NRS	SFS	BNSS	TSQ	Pedometer	MST
Parameter	Beta (S.E.)	Beta (S.E.)	Beta (S.E.)	Beta (S.E.)	Beta (S.E.)	Beta (S.E.)	Beta (S.E.)
Fixed effects							
Time factor							
T0	56.96 (1.79)	91.35 (2.51)	125.62 (2.84)	1.11 (0.12)	14.77 (0.43)	9344.52 (659.11)	9.76 (0.44)
T1effect	4.13 (2.31)	7.76 (2.17)	6.16 (2.30)	0.24 (0.12)	-0.02 (0.50)	-1594.19 (1035.17)	-0.31 (0.32)
*X* ^*2*^	5.421	3.372	0.128	1.523	1.725	0.000	0.912
T1 effect BEATVIC	-5.67 (3.30)	-7.15 (3.11)	-7.01 (3.30)	-0.22 (0.17)	-0.39 (0.72)	1658.45 (1432.21)	0.85 (0.43)
*X* ^*2*^	-2.914	5.192	4.437	1.657	0.281	1.335	3.768
T2 effect	-1.86 (2.46)	3.39 (2.32)	5.26 (2.46)	0.07 (0.13)	0.77 (0.57)	n.a.	n.a.
*X* ^*2*^	5.421	3.372	0.128	1.523	1.725	n.a.	n.a.
T2 effect BEATVIC	2.72 (3.44)	-2.13 (3.24)	-5.17 (3.45)	0.24 (0.17)	-0.75 (0.77)	n.a.	n.a.
*X* ^*2*^	0.625	0.429	2.226	1.836	0.360	n.a.	n.a.
Random effects							
*Variances of*							
Level 2—intercept	46.28 (14.35)	225.66 (37.73)	305.22(49.52)	0.46 (0.08)	3.70 (0.90)	0.00 (0.00)	13.11 (2.05)
Level 1—residual	120.55 (13.59)	101.79 (11.65)	114.64 (13.14)	0.29 (0.03)	3.97 (0.56)	19114732 (2164317)	1.01 (0.19)

Note.

MANSA: Manchester Short Assessment of Quality of Life, NRS: National Recovery Scale, SFS: Social Functioning Scale, BNSS: Brief Negative Symptom Scale, TSQ: Trauma Screening Questionnaire, MST: Modified Shuttle Test. All effects were non-significant with *p*-values above 0.05.

### Per protocol analysis for continuous outcomes

Per protocol analyses including data of participants that attended ≥ 75% of the sessions did not show different results on the continuous outcomes in multilevel analyses. In addition, no time * condition effects were found.

## Discussion

The current study compared the effect of a preventive body-oriented resilience therapy ‘BEATVIC’ with an active control condition in a multicenter randomized controlled trial. The aim of this study was to assess the short-term effects of BEATVIC on victimization, and its risk factors, in people with a psychotic disorder. More than half of the participants demonstrated a reduction in victimization or reported no new incidents (60.5% BEATVIC vs 62.9% befriending). However, there was no significant difference between BEATVIC or befriending. No differences were found in primary and secondary outcomes between BEATVIC and befriending at post treatment and six months follow-up.

For both conditions, victimization incidents either reduced over time, or did not occur, with very few new victimization incidents occurring during the period of the intervention and the 6 months follow-up. It is possible that both BEATVIC and befriending act on factors which allow the risk of victimization to be reduced or stabilized. A recent RCT showed that befriending is effective in increasing social contacts on the short term in people with schizophrenia [49]. An increase in social contacts may also indirectly contribute to reducing the risk of victimization, given that victimization in people with psychosis is correlated with poorer social functioning [50]. Possible differentiating effects of BEATVIC in comparison to befriending in the long-term will be examined at 18-month and 30-month follow-up. The long-term follow up will also demonstrate whether BEATVIC can prevent other types of victimization (e.g. sexual assault), given that most victimization incidents on the short-term in this study reflected psychological aggression.

There was no improvement on the risk factors of victimization in BEATVIC as compared to befriending on the short term. This contrasts the feasibility study (N = 24), in which participants subjectively indicated a positive effect of BEATVIC on recognizing others’ boundaries, identifying and setting boundaries, self-esteem, faith in own strength, confidence, recognizing dangerous situations and risk of victimization [13]. In addition, in the fMRI sub study including 27 participants, brain activation was examined during two social cognition tasks [46]. Findings demonstrated increased involvement of the salience network in processing angry and fearful faces in BEATVIC participants compared to befriending, suggesting an increased alertness for potentially dangerous faces. As this change was not found in the behavioral data in this study, it could be argued that short-term effects of BEATVIC are restricted to basic neuropsychological mechanisms (fMRI study) and subjective reports (feasibility study).

Previous research investigating body and movement oriented therapies demonstrated improvements on self-esteem, social interaction skills and psychiatric symptoms in people with a psychotic disorder [9, 51, 52]. Studies suggest that martial-arts training like kickboxing, could have a positive effect on aggression regulation and social interaction [53–55]. The aforementioned studies differ from the current RCT, in terms of: 1) a higher frequency (2 or 3 weekly sessions) and/or a longer duration (one year or longer) of interventions, 2) samples (children and/or adults from the general population), and 3) measures (observer rated and/or qualitative interviews/questionnaires).

Previous studies on exercise interventions reporting beneficial effects mostly investigated interventions with a higher frequency and duration. An average weekly exercise frequency of at least 2 times a week might be the minimum to achieve beneficial effects [46, 56]. BEATVIC consisted of weekly sessions and included therapeutic elements in addition to kickboxing exercises, and was therefore less intense than the aforementioned interventions. Another possible explanation a lack of effect, relates to the heterogeneity of the group of participants and the broad range of risk factors that were addressed in BEATVIC. For sub-assertive individuals experiencing difficulties standing up for themselves, exercises were aimed to increase empowerment and assertiveness. For others, exercises were aimed at aggression regulation problems and aimed at participants that may evoke conflicts ultimately leading to victimization. To promote change in this broad and heterogenic patient group, BEATVIC could be tailored to a specific victimization type, targeting fewer risk factors more efficiently, more frequently (>2 times per week) and for a longer duration.

Participants were not specifically selected based on risk factors of victimization, as the patient group has a-priori increased chances of victimization. When comparing the baseline rates of victimization in our sample with rates of victimization in the general population, our sample was 3–4 times as likely to report physical assault and 2 times as likely to report sexual assault in the past five years [57]. However, it is possible that participants who were not prone to victimization at that point in time were also included. Indeed, more than half of responders in both conditions did not report any incidents of victimization, and baseline data show that on average, participants did not have deviant scores on self-esteem, social cognition, insight, self-stigma, and aggression regulation. Furthermore, as our exclusion criteria prohibited people with severe psychotic symptoms or substance dependence (risk factors for victimization [2]) from taking part, the study could have precluded the most vulnerable patients. However, we chose to exclude patients with these vulnerabilities, because these risk factors also hinder structural participation in groups. For future research, it is recommended to examine if BEATVIC can also be effective for dual-diagnosis groups (e.g. psychotic disorder with comorbid substance dependence) as this group seems particularly prone to victimization [2]. Furthermore, replicating the current study using a larger sample size could also yield more power to detect differences between individuals vulnerable and not vulnerable to victimization, after taking part in the BEATVIC group sessions.

The current study made use of self-report quantitative measures, whereas previous studies investigating body-oriented therapies focused on observer rating measures and qualitative questionnaires. A previous study examining a martial arts intervention (similar to BEATVIC in terms of time/duration, and diagnostic characteristics) found an increase in self-control and sense of empowerment based on qualitative interviews [58]. The BEATVIC feasibility study showed promising results based on a semi-structured questionnaire and group evaluations [13]. Perhaps these subjective benefits are not easily objectified with standardized questionnaires. Common criticism concern the requirement of needing insight in one’s own behavior, the potential induction of social desirability bias, or biases related to timing [59]. Regardless, all measures used in the current study have good psychometric properties and were carefully chosen.

### Strengths and limitations

Although individuals with a psychotic disorder have a higher risk to falling victim to a crime than people from the general population [1], no evidence-based intervention aimed at preventing victimization in individuals with psychosis is available. The current study is the first to develop and test an intervention aimed at preventing victimization for individuals with a psychotic disorder in a randomized controlled trial.

The current study also has limitations. The sample was not selected on the basis of victimization risk, and is heterogenous in terms of the type of crime the participants were victim of. The intervention was carried out using a lower frequency and duration in comparison to previous studies, and the study relied mostly on self-report outcomes.

### Implications and future directions

Findings of the current study demonstrate that BEATVIC was not more effective at preventing victimization for individuals with a psychotic disorder than an active control condition at short term follow-up. However, the fact that victimization incidence decreased over time in both conditions is promising and analysis of the follow-up data is warranted to investigate long term effects on preventing victimization.

For future studies we recommend examining possible victimization types, enabling personalized predictions of who is at risk and tailor the intervention to these victimization types. Furthermore, proximal outcomes are recommended for future studies, such as non-verbal measures to test differences in arousal in social interactions and/or experience sampling to measure changes over time in interactions in daily life. For example, the PsychoMotor Diagnostic Instrument (originally developed for post-traumatic stress, PMDI [60]) may be suitable, as this assesses expected non-verbal outcomes of our intervention (e.g. stress level, physical fitness and vitality, trust and impulsive aggressive behavior). Last, it is recommended to replicate the current study using larger sample sizes and including other psychiatric populations as well, in order to further examine the effectiveness of the BEATVIC group sessions.

## Supporting information

S1 Checklist BEATVIC CONSORT 2010 checklist. (DOC)

S1 File Study protocol BEATVIC. (PDF)
