# Supplementary material for: BEATVIC, a body-oriented resilience therapy for individuals with psychosis: Short term results of a multi-center RCT
Source: PLoS One. 2022 Dec 21;17(12):e0279185. doi: 10.1371/journal.pone.0279185 (PMC9770373; doi:10.1371/journal.pone.0279185)
Supplement: S1 File — (PDF) [file pone.0279185.s002.pdf]

Beat Victimization!
Psychomotor assertiveness training with elements of kickboxing for people with psychotic disorders

(November 2016)

**Short title**	**Beat Victimization!**
**Version**	**4**
**Date**	**September 2015**
**Project leader**	**Dr. Jooske van Busschbach**<br>**Universitary Center Psychiatry, UMCG**<br>**Hanzeplein 1, Groningen**<br>**Tel. 050 361 2069**
**Principal investigator(s)**	**Dr. Marieke Pijnenborg (GGZ Drenthe/RUG)**<br>**Dr. J.T. van Busschbach (UMCG/Windesheim)**<br>**Prof. A. Aleman (RUG/NIc)**<br>Uitvoerend onderzoekers<br>**B. de Vries MSc (RUG)**<br>**E. van der Stouwe MSc (UMCG)**
**Sponsor**	**Rijksuniversiteit Groningen/UMCG**
**Subsidising party**	**Nederlandse organisatie voor Wetenschappelijk Onderzoek (NWO)**
**Independent expert(s)**	**Dr. Jacqueline Quak**<br>**Tel: 050-3612065**
**Laboratory site**	**GGZ Drenthe, GGZ Friesland, Lentis, UCP, Dimence, Arkin**

TABLE OF CONTENTS

1. INTRODUCTION AND RATIONALE 7
2. OBJECTIVES 10
3. STUDY DESIGN 10
4. STUDY POPULATION 11
   - 4.1 Population (base) 11
   - 4.2 Inclusion criteria 11
   - 4.3 Exclusion criteria 11
   - 4.4 Additional exclusion criteria for the fMRI-part 11
   - 4.1 Sample size calculation 12
5. TREATMENT OF PARTICIPANTS 12
   - 5.1 Investigational intervention 12
     - 5.1.1 Modules 13
     - 5.1.2 Session procedure 15
6. METHODS 15
   - 6.1 Study parameters/endpoints 15
     - 6.1.1 Selection parameters 15
     - 6.1.2 Main study parameters/end point 16
     - 6.1.3 Main study parameters/end point MRI-part 20
     - 6.1.4 Covariates 20
   - 6.2 Randomisation, blinding and treatment allocation 21
   - 6.3 Study procedures 21
   - 6.4 Withdrawal of individual participants 23
   - 6.5 Replacement of individual participants after withdrawal 23
7. SAFETY REPORTING 24
   - 7.1 Section 10 WMO event 24
   - 7.2 Adverse events (AEs) 24
8. STATISTICAL ANALYSIS 25
   - 8.1 Descriptive statistics 25
   - 8.2 Analysis 25
   - 8.3 fMRI Analysis 25
   - 8.4 Regulation statement 26
   - 8.5 Recruitment and consent 26
   - 8.6 Benefits and risks assessment, group relatedness 27
   - 8.7 Compensation for injury 27
   - 8.8 Incentives 28
9. ADMINISTRATIVE ASPECTS, MONITORING AND PUBLICATION 28
   - 9.1 Handling and storage of data and documents 28
   - 9.2 Amendments 28
   - 9.3 Annual progress report 28
   - 9.4 End of study report 29

LIST OF ABBREVIATIONS AND RELEVANT DEFINITIONS

**ABR**	ABR form, General Assessment and Registration form, is the application form that is required for submission to the accredited Ethics Committee (In Dutch, ABR = Algemene Beoordeling en Registratie)
**AE**	Adverse Event
**BNSS**	Brief Negative Symptom Scale
**CCMO**	Central Committee on Research Involving Human Subjects; in Dutch: Centrale Commissie Mensgebonden Onderzoek
**IOA**	Inventarisatielijst omgaan met anderen
**ISMI**	Internalized Stigma of Mental Ilness
**IVM**	Integrale veiligheidsmonitor
**MANSA**	Manchester Short Assessment of Quality of Life
**METC**	Medical research ethics committee (MREC); in Dutch: medisch ethische toetsing commissie (METC)
**MST**	Modified Shuttle Test
**NWO**	Nederlandse organisatie voor Wetenschappelijk Onderzoek
**PANSS**	Positive And Negative Symptoms Syndromes
**PMT**	Psychomotor therapy
**PTSD**	Post-Traumatic Stress Disorder
**RCT**	Randomized Controlled Trial
**SERS-SF**	Self-Esteem Rating Scale – Short Form
**SIG**	Schaal Interpersoonlijk Gedrag
**SFS**	Social Functioning Scale
**Sponsor**	The sponsor is the party that commissions the organisation or performance of the research, for example a pharmaceutical company, academic hospital, scientific organisation or investigator. A party that provides funding for a study but does not commission it is not regarded as the sponsor, but referred to as a subsidising party.
**CTS2**	Revised Conflict Tactics Scale
**TSQ**	Trauma Screening Questionnaire
**Wbp**	Personal Data Protection Act (in Dutch: Wet Bescherming Persoonsgevens)
**WMO**	Medical Research Involving Human Subjects Act (in Dutch: Wet Medisch-wetenschappelijk Onderzoek met Mensen
**ZECV**	Self-Expression and Control

SUMMARY

Rationale

Contrary to what is generally thought, people with psychotic disorders are more likely to be a victim of a crime, than an offender. Research has revealed several factors to be associated with victimization in patients with psychotic disorder. Impaired social cognition, decreased insight and problems in aggression regulation may lead to inadequate social behavior which increases the risk of victimization. In turn victimization can increase self-stigma resulting in reduced self-esteem, empowerment, and assertiveness, making patients more vulnerable for victimization. In addition, the traumatic experience of the victimization could induce strong physiological responses to external stimuli resulting in fear regulation problems. Consequently, becoming a victim significantly enhances risk for revictimization. The aim of this study is to investigate a new intervention which addresses the risk factors that may help prevent victimization of people with psychotic disorders.

Objective:

The proposed study aims to investigate the effects of a psychomotor assertiveness training by means of a multicentre randomized controlled trial (RCT). Effects will be examined at the behavioural level by means of questionnaires and interviews, and at the cerebral level with an fMRI task. The effect on actual victimisation will be investigated as a long term outcome, because the prevalence of incidents is low thus we expect that effects of the intervention will only be visible after a longer period of time. Short term effects on risk factors of victimization (e.g. social cognition, self-stigma, assertiveness, self-esteem, aggression regulation, social behaviour) will be examined, since these are direct targets of the intervention. We expect that the risk factors will mediate the effect between the training and victimization.

Study design

To test the effectiveness of the intervention, an RCT with a pretest-posttest and follow-up will be performed.

Study population

120 participants will be recruited from GGZ Drenthe, GGZ Friesland, GGZ Centraal, Lentis, UCP, Arkin and Dimence. Twenty-two participants per condition will be included in the fMRI part of the study. Onsite therapists will select the potentially eligible patients using seven screening questions.

Intervention

A psychomotor assertiveness training with elements of kickboxing aims to decrease the risk of victimization. The training consists of 20 weekly group training sessions divided into five modules (see 5.1.1 for a description of the modules). Each session takes 75 minutes. Groups contain a maximum of ten participants.

Main study parameters/endpoints

Primary study outcome is the long term effect of the psychomotor assertiveness training with elements of kickboxing on victimization, as measured by two questionnaires which examine whether an individual has been victim of a crime. We expect that this effect will be mediated by risk factors of victimization (e.g. social cognition, internal stigma, assertiveness, self-esteem, aggression regulation, social behaviour), as measured by both behavioural and cognitive measures such as interviews, questionnaires and a neuropsychological test, and cerebral measures such as fMRI paradigms. Secondary long term outcome measures are quality of life, recovery, social participation, negative symptoms and coping with trauma.

Nature and extent of the burden and risks associated with participation, benefit and group relatedness

For all participants, the selection assessment will take approximately 60 minutes, the pre-test will take approximately 160 minutes, the pos-test will take approximately 180 mintues, the first follow-up assessment takes 180 mintues and the second and third follow-up assessment will take approximately 110 minutes. For the participants in the fMRI study, the pre- and post MRI appointments will both take approximately 90 minutes. For participants randomised in the experimental group, the intervention consists of 20 sessions of 75 minutes. For participants randomised in the control group, 20 befriending sessions are organised. In the media material arts are sometimes associated with aggressive behavior. However, most research does not support this finding or shows opposite results. It is unlikely that kickboxing in itself increases aggressive behavior. Based on a recent feasibility study we don’t expect any adverse events to happen for this reason and consider no such risks to be associated with participation.

1. INTRODUCTION AND RATIONALE

Contrary to what is generally thought, people with psychotic disorder are more likely to be a victim of a crime, than an offender (Brekke, Prindle, Bae, & Long, 2002; Dean et al., 2007). Prevalence rates vary between studies because of the differences in e.g. population samples, instruments, and time period covered. For example, between 16% (Walsh et al., 2003) to 33% (Bengtsson-Tops & Ehliasson, 2012) of people with psychotic disorders indicated they had been a victim of a violent crime in the preceding year. While looking at lifetime prevalence percentages range from 25% (Short, Thomas, Luebbers, Mullen, & Ogloff, 2013) to 78.8% (Dolan, O'Malley, & McGregor, 2013) depending on the definition of victimization and the time frame examined. In a recent Dutch study of Kamperman et al. (2014) 47% of the severe mentally ill outpatients reported that they had been a victim of a crime in the past year. Compared to the general population, people with severe mental illness reported fourteen times more incidents regarding personal crime such as sexual harassment or assault, threats of violence, and physical assault.

Victimization can have a major impact on the lives of already vulnerable patients, and ultimately entails high costs for society (Choe, Teplin, & Abram, 2008). The increasing deinstitutionalization of the mental health care system puts an increasing demand on the assertiveness of people with psychotic disorder, possibly enhancing the victimization risk in the future (Short et al., 2013). At this moment there is no evidence-based intervention that can decrease the victimization risk for people with psychotic disorder (Weeghel, Kamperman, Vries, Plooy, & Mulder, 2009), while both patients and caregivers indicate the need for one (Busschbach, Wiersma, & Slooff, 2012). Therefore, we are developing an intervention that aims to prevent victimization.

Before the development of the intervention we contemplated the underlying processes. Research has revealed several factors to be associated with victimization in patients with psychotic disorder. For example, alcohol and drug use (Chapple et al., 2004), homelessness (Walsh et al., 2003), perpetration (Honkonen, Henriksson, Koivisto, Stengård, & Salokangas, 2004), lack of meaningful daily activity (Fitzgerald et al., 2005), comorbid personality disorder (Dean et al., 2007), severe symptoms (Brekke, Prindle, Bae, & Long, 2001), and positive symptoms are all associated with an increased risk of victimization (Cohen, Palekar, Barker, & Ramirez, 2012). Not all of these factors can be readily addressed in one psychomotor intervention. For this reason, we developed a model in which we selected some of the important risk factors that may lead to victimization and are amendable to change (see Figure 1).

The first risk factor we would like to address is social cognition (DePrince, 2005). Compared to the general population people with a psychotic disorders have more difficulty with recognizing facial expressions (Evangeli & Broks, 2000), body language (Phillips & Seidman, 2008), and

emotional prosody (Hoekert, Kahn, Pijnenborg, & Aleman, 2007). This may complicate the judgment of risky social situations. For instance, they may not see it coming when one of their friends is going to be aggressive.

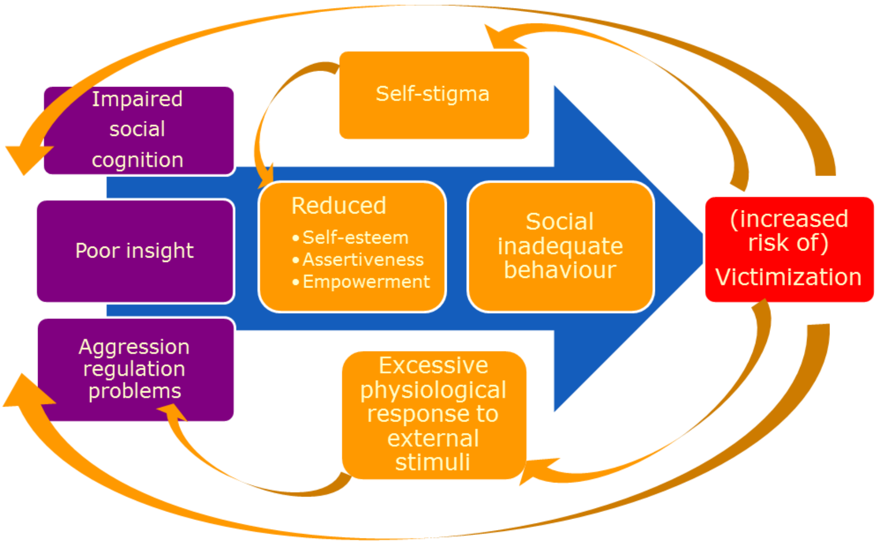

Figure 1: Victimization model

The second factor is insight, according to Ekinci & Ekinci (2013) poor insight is one of the pathways to aggression in schizophrenia. Poor insight in own psychotic symptoms may result in aggressive behavior creating dangerous situations for others as well as for themselves (Hiday, Swartz, Swanson, Borum, & Wagner, 1999). In accordance, problems in aggression regulation are associated with violent behavior in patients with a psychotic disorder (Witt, van Dorn, & Fazel, 2013). For this reason aggression regulation is the third risk factor that will be addressed. By addressing these three risk factors the intervention may improve social behavior and decrease victimization risk.

Another factor that may play a role in victimization is self-stigma. There is a lot of prejudice beliefs about mental illnesses. Most patients are aware of these and believe some of them (Brohan, Elgie, Sartorius, & Thornicroft, 2010). Victimization can increase self-stigma (Horsselenberg, Pijnenborg & van Busschbach, submitted), resulting in low self-efficacy (Kleim et al., 2008), low self-esteem and reduced empowerment (Livingston & Boyd, 2010). Consequently, people may not stand up for themselves in social situations and are therefore more prone to become a victim (Egan & Perry, 1998), which in turn increases self-stigmatic ideas and feelings of helplessness making this a self-enforcing process. Victimization not only affects self-stigma but also reinforce the other risk factors mentioned in the victimization model, increasing the chance of revictimization.

In addition, the traumatic experience of being a victim could lead to a strong physiological response to external stressors (Peri, Ben-Shakhar, Orr, & Shalev, 2000) and a compromised

inhibitory control (Falconer et al., 2008). In accordance, people who suffer from trauma or stress could react more aggressive in social situations (Makin-Byrd, Bonn-Miller, Drescher, & Timko, 2012; Volavka & Citrome, 2011). This aggressive response may elicit conflicts, again putting people at risk for victimization. Ultimately, people can become trapped in a downward spiral and need help to emerge from this vicious victimization cycle.

To address these specific risk factors we developed a psychomotor assertiveness training with elements of kickboxing. We chose a psychomotor approach because the results of body and movement oriented therapies look promising for people with a psychotic disorder (Röhricht, 2009; Röhricht, Papadopoulos, Suzuki, & Priebe, 2009). More research is warranted but review studies of Holley et al. (2011) and Malchow et al. (2013) show that physical exercise therapy, varying from swimming, football to cardiovascular exercise could have a positive effect on self-efficacy, self-esteem, social interaction skills, and positive and negative symptoms. These effects show an overlap with the risk factors in the victimization model and could help to decrease victimization risk. The same is true for assertiveness training. Assertiveness training can improve self-esteem, perceived control, assertiveness, and self-efficacy (Brecklin, 2008). Improving these factors may have a positive effect on social behavior and decrease victimization risk (see Figure 1 victimization model).

The intervention includes martial arts techniques. Several studies suggest that martial arts could have a positive effect on e.g. aggression regulation and social interaction (Elling, Wisse, & Berk, 2010; Lakes & Hoyt, 2004; Twemlow et al., 2008; Zivin et al., 2001). Our main technique is kickboxing since this is a sport that integrates both a strong focus on regulatory techniques with physical exercises. Furthermore, basic kickboxing techniques are achievable for everyone regardless of someone's physical condition. The active exercises in kickboxing may contribute to a pleasurable sportive experience in the sessions and give enough opportunities to address the risk factors. In the media, martial arts are sometimes associated with aggressive behavior. Most research does not support this finding or shows opposite results (Elling et al., 2010; Vertonghen & Theeboom, 2010). Some studies show that children or adolescents who practice fighting sports are more aggressive, compared to people who practice other types of sport. However several confounders play a role here as these studies are cross-sectional: more aggressive youths choose for example kickboxing because of its hard reputation (Elling et al., 2010; Lamarre & Nosanchuk, 1999; Vertonghen & Theeboom, 2012). Other factors that may play a role in these aggressive outcomes are a negative 'macho' environment at the gym (Eddehbia & Van der Putte, 2009; Endresen & Olweus, 2005), and competitive and performance oriented guidance (Nosanchuk & MacNeil, 1989). Kickboxing in a non-competitive non-commercial setting has been shown to have positive psychological effects and not enhance everyday aggression (Elling et al., 2010). In conclusion, it is unlikely that kickboxing in itself increases aggressive behavior.

2. OBJECTIVES

In preparation of the proposed multicentre randomized controlled trial (RCT), the psychomotor assertiveness training was tested in a feasibility study (n=23, dossier number NL49520.042.14, ABR number 49520). Subsequently, the intervention protocol has been evaluated and improved. The proposed study aims to investigate the effects of a psychomotor assertiveness training by means of a multicentre randomized controlled trial (RCT). Effects will be examined at the behavioural level by means of questionnaires and interviews, and at the cerebral level with an fMRI task. The effect on actual victimisation will be investigated as a long term outcome, because the prevalence of incidents is low thus we expect that effects of the intervention will only be visible after a longer period of time. Short term effects on risk factors of victimization (e.g. social cognition, self-stigma, assertiveness, self-esteem, aggression regulation, social behaviour) will be examined, since these are direct targets of the intervention. We expect that the risk factors will mediate the effect between the training and victimization.

3. STUDY DESIGN

The proposed study is an RCT with a pretest, two posttests and two follow-up assessments. A flowchart of the design is presented in Figure 2. For a detailed description of the recruitment and consent procedure see 7.5 and for the study procedure see 5.3.

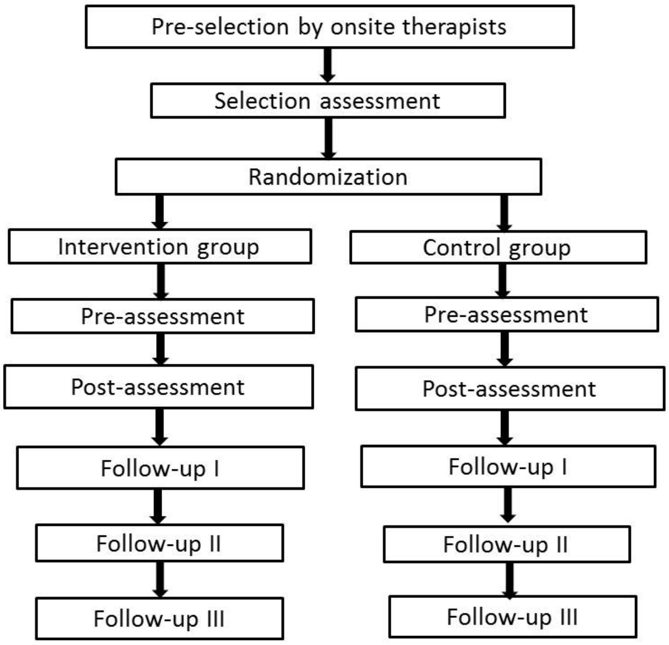

Figure 2: Flow chart of study design

STUDY POPULATION

4. STUDY POPULATION

4.1 Population (base)

Participants are individuals with a diagnosis in the psychotic spectrum and will be recruited from GGZ Drenthe, GGZ Friesland, GGZ Centraal, Dimence, Arkin, Lentis and UCP. Both in- and outpatients can be included in the study.

4.2 Inclusion criteria

In order to be eligible to participate in this study, a participant must meet all of the following criteria:
- A diagnosis in the psychotic spectrum, according to DSM-IV-TR criteria, verified by MiniScan;
- Being able to give informed consent;
- 18 years or older;

4.3 Exclusion criteria

A potential participant who meets any of the following criteria will be excluded from participation in this study:
- Florid psychosis (mean positive symptoms > 5 measured by PANSS);
- Substance dependence (not substance abuse) of alcohol, marijuana, opiates, stimulants and cocaine, verified by Miniscan.
- Estimated IQ < 70, onsite therapist decides if the patients intelligence is sufficient for participation.
- Co-morbid neurological disorder, verified by onsite therapist.
- Co-morbid personality disorder, verified by onsite therapist.
-Pregnancy.
- Current participation in martial arts classes.

4.4 Additional exclusion criteria for the fMRI-part

All participants will fill out a questionnaire covering safety aspects and exclusion criteria of research in a 3 Tesla magnetic field and MRI environment. Exclusion criteria are:

- MR incompatible implants in the body (such as ear prosthesis or other metal implants)
- Any risk of having metal particles in the eye, due to manual work without proper eye protections
- Tattoos containing red pigments
- (Suspected) pregnancy
- Claustrophobia

- Refusal to be informed (by notifying the patients physician) of structural brain abnormalities that could be detected during the experiment

4.1 Sample size calculation

Sample size was calculated using the computerprogram SPSS Sample Power. In order to find a medium effect size (0.50) with two-sided alpha=0.05, power=0.80 and taking 25% drop-out into account, 60 participants per group are needed. This means a total sample size of 120 participants.

Sample size in fMRI studies is usually not determined by power analyses, but by experiences with similar protocols. Exact power calculations are difficult because of the complex 'mass univariate' nature of brain imaging data as well as the difficulty to estimate effect sizes in different regions of interest beforehand. Previous studies suggest that in general, 20 participants are necessary per group to achieve sufficient power and acceptable reliability (Monti, 2011; Thirion et al., 2007). Therefore, in the fMRI-part of the proposed study, 22 participants will be included per group.

5. TREATMENT OF PARTICIPANTS

5.1 Investigational intervention

As described in chapter 1 there are different kinds of risk factors that may play a role in the victimization of patients with a psychotic disorder. Based on these risk factors (see Figure 1 for the victimization model) we developed a psychomotor assertiveness training with elements of kickboxing. The intervention is developed by a project team that consists of the following people:

-*Marieke Pijnenborg*, associated professor at the University Groningen, specialized in psychotic disorder. She is also affiliated as head of the department of scientific research and diagnostics, and cognitive behaviour therapist at the department of psychotic disorders at GGZ-Drenthe.

-*Jooske van Busschbach*, senior researcher at the University Medical Centre Groningen, specialized in effect studies of psychosocial interventions in psychiatry. She is also lector at the Windesheim University of applied science with psychomotor therapy and the interaction between exercise and physical experience with health and welfare as area of expertise.

- *Clement Waarheid,* psychomotor therapist at the department of psychotic disorders at GGZ-Drenthe. He has experience in developing psychomotor interventions and gives psychomotor kickboxing training for people with a psychotic disorder.

- *Stef Poel and Aniek Meerdink,* both working as expert by experience for the psychotic department of GGZ-Drenthe. They are experienced kick boxers themselves and have given psychomotor kickboxing training for people with a psychotic disorder.

- *Erwin van der Helm,* fighting sports expert with experience in developing self-defence training. Van der Helm has his own fighting school where he works in cooperation with psychologists and he is an experienced kickboxing trainer.
- *Bertine de Vries,* PhD student at the department of clinical psychology of the University of Groningen. One of the coordinating researchers of the Beat victimization project.
- *Elise van der Stouwe,* PhD student at the University Medical Centre Groningen. One of the coordinating researchers of the Beat victimization project.
- *André Aleman*, is professor of Cognitive Neuropsychiatry at the Department of Neurosciences of the UMCG and the Department of Psychology of the University of Groningen.

In addition, we composed an advising committee of people who have both fighting sports experience and expertise in the field of psychiatry, psychology or psychomotor training. This committee critically reviewed the intervention protocols and provided feedback and suggestions for improvement.

The intervention consists of 20 weekly group training sessions of 75 minutes divided into five modules (see 4.1.1 for a description of the modules). Research shows that psychomotor therapy can have an effect on the mental health of patients with schizophrenia, if they have weekly sessions of one or two hours for three or six months (Marzolini, Jensen, & Melville, 2009; Scheewe et al., 2013), thus one session per week for five months should be sufficient to sort out an effect. Each session includes a warming-up, cooling-down, carry out the therapeutic martial arts exercises, and a group talk about the addressed themes (see 5.1.2 for the session procedure). The intervention has been evaluated and improved by means of a feasibility-study. Based on the feedback of participants as well as the trainers, the fighting expert and the researchers, some exercises have been adapted. For example, intensity, duration or techniques are changed in order to improve effectiveness.

The manual of the training and theoretical background information about the exercises and procedures are provided in appendix F2. All the sessions will be given by a psychomotor trainer and an expert by experience trained by the team that developed, gave and evaluated the training during the feasibility-study. The training consists of four sessions of 2,5 hours during which the most important exercises are trained and most important information is discussed. When the psychomotor assertiveness training has started, monthly supervision will be provided by the study investigators.

5.1.1 Modules

<u>Module 1 Introduction</u>

In the first module the participants will be introduced to each other and learn more about the session procedures. The exercises and information focuses on self-stigma, safety, respect for each other, and how to establish and set boundaries. In addition, participants will monitor the tension level in their body, during and after exercises, and learn how to reduce tension. All exercises are done with the help of six basic kickboxing punches and kicks, and a basic fighting stance.

Module 2 Recognizing danger

The second module focuses on social cognition and insight. In addition to exercises from the first module that are repeated new exercises are offered regarding the recognition of emotion in facial expressions, prosody, and body language. Participants will learn the importance of considering other people's perspectives and how to verify their own. The techniques used in this module are blocking and deflection of the techniques learned in the first module. In addition, there are non-violent de-escalation techniques learned as an example of what a person could do in a threatening situation.

Module 3 How others see me

In the third module the participants will be practicing perspective taking To address this topic the participants will explore what kind of non-verbal signals they send out and find out how they react in threatening situations. Is their non-verbal reaction helpful or does it bring them in trouble? Furthermore, participants will learn how they can convincingly protect their boundaries and what signals to send out in threatening situations. The techniques used in this module are a combination of elements learned in the previous modules plus new basic kickboxing techniques.

Module 4 Aggression regulation

The focus of the fourth module is on aggression regulation. Ever since the first module the participant are paying attention to the tension in their body throughout each session. In this module participants will focus on anger and aggression: What are the bodily signals that their anger or stress is escalating and how does this affect their reactions to external stimuli? Participants will learn how to step back and reduce tension or stress. We will not introduce new kickboxing techniques but concentrate on the technique, power and speed of the ones already learned.

Module 5 Repeat and combine

In the fifth module exercises and topics from previous modules are combined and repeated when necessary. Furthermore, participants are stimulated to continue martial arts in a regular

sporting gym. A guest trainer from a local fighting school is invited to give a lesson of 30 minutes, making the step to a regular school more accessible for the participants.

5.1.2 Session procedure

There are 20 sessions of 75 minutes each. Table 1 shows the main program for each session. For a detailed description and background information about the program see appendix F2.

Table 1: session procedure

Time in minutes	Program
10	1. Greeting ceremony and warming-up
30	2. Thematic exercises
15	3. Repetition and extra exercises
10	4. Cooling-down and greeting ceremony
10	5. Discussion

6. METHODS

6.1 Study parameters/endpoints

6.1.1 Selection parameters

For the selection assessment we will use the PANSS to see whether the patient's level of positive symptoms is not too high for participation (see 3.3 Exclusion criteria and 5.1.1 main study parameters). To verify the DSM diagnosis and exclude alcohol and drugs dependence we will use the mini Schedules for Clinical Assessment in Neuropsychiatry (miniSCAN; WHO, 1992, 1999 vertaling Giel & Nienhuis 2000).

Table 3: other study parameters

Parameter	Instrument	Duration	Type
Symptoms	PANSS	minutes	Semi structured interview
Diagnosis	MiniSCAN	30 minutes	Structured interview

Positive and Negative Symptom Scale

The Positive and Negative Syndrome Scale (PANSS) is administered to monitor the changes in primary symptoms. The PANSS is a 30 item rating scale based on chart review and a semi-structured interview. It is widely used for assessing positive, negative and cognitive symptoms (Kay, Flszbein, & Opfer, 1987).

MiniSCAN

The miniSCAN is a short version of the Schedule for Clinical Assessement in Neuropsychiatry (SCAN 2.1) and consists of a structured interview questions regarding axis-I symptoms of the DSM-IV (Damhuis, van Megen, Peeters, & Vollema, 2011).

6.1.2 Main study parameters/end point

The main study parameters and subsequent instruments used in this study are shown in table 4.

Table 4: Main study parameters

Parameter	Instrument	Duration	Type
Primary outcome measures			
Victimization	IVM	10 minutes	Self-rating
	CTS2	20 minutes	Self-rating
Mediating factors			
Social cognition	Faux pas task	20 minutes	Neuropsychological task
	Wall of faces task**	20 minutes	fMRI task
	Emotional faces paradigm**	15 minutes	fMRI task
Self-expression and control	ZECV	10 minutes	Self-rating
Internalised stigma	ISMI	15 minutes	Self-rating
Social behavior	SIG	15 minutes	Self-report
Self-esteem	SERS-SF	10 minutes	Self-rating
Secondary outcome measures			
Quality of life	MANSA	5 minutes	Self-rating
Trauma	TSQ	5 minutes	Self-rating
Recovery	NHS	10 minutes	Self-rating
Societal participation	SFS	20 minutes	Self-report
Negative symptoms	BNSS in addition to PANSS	30 mintues	Semistructured interview
Fitness	MST	15 minutes	Physical test
Physical activity	Accelerometer	1 week	Physical measure

*IVM= Integrale Veiligheidsmonitor

CTS2= Revised Conflict Tactics Scale

ZECV= Self-Expression and Control Scale

ISMI= Internalized Stigma of Mental Ilness

IOA= Inventarisatie Omgaan met Anderen

SERS-SF= Self-Esteem Rating Scale – Short Form

BNSS= Brief Negative Symptom Scale

MANSA= Manchester Short Assessment of Quality of Life

TSQ= Trauma Screening Questionnaire

NHS= Nationale herstelschaal

SFS= Social Functioning Scale

** In case of participation in the MRI-substudy

Integrale Veiligheidsmonitor

The Integrale Veiligheidsmonitor (IVM) is a Dutch crime and victimization survey that resembles the international crime victimization survey (CBS, 2009). We will use the victimization subscale (Victrom: Kamperman et al., 2014) and the subscales about safety perception in general and in one's neighborhood. Participants will complete the IVM pre- and post- treatment as well as during both follow up assessments, since we are particularly interested in victimization in the long term. The time-frame in the questions will be adapted, in order to investigate victimization between two subsequent assessment points. At baseline, the time-frame consists of the past five years. At both post assessments, victimization of past six months will be examined. At follow-up victimization of the past year will be assessed.

Revised Conflict Tactics Scale

The revised Conflict Tactics Scale (CTS2) measures to what extent the respondent is involved in a psychological or physical attack and the use of reasoning or negotiation to deal with conflicts (Straus, Hamby, Boney-McCoy, & Sugarman, 1996). The CTS2 is a widely used instrument in partner violence research and consist of 39 behavior items. Participants report how many times, ranging from 1 'has never happened' to 8 'more than twenty times' the items apply on themselves or their partner in a given time period (Vega & O'Leary, 2007). Since we are interested in a broader range of social interactions we changed the questions from 'partner' to 'someone'. For example 'I kicked my partner' and 'My partner did this to me' is changed in 'I kicked someone' and 'Someone did this to me'. The CTS2 assesses more subtle forms of victimization, therefore this instrument is a good addition to the IVM.

Social Functioning Scale

The Social Functioning Scale (SFS) assesses social functioning and social participation. It consists of 78 items divided by 7 subscales: social engagement/withdrawal, interpersonal communication, independence-competence, independence-performance, recreation, pro-social behavior and employment. The SFS is reliable, valid, sensitive and responsive to change (Birchwood et al. 1990)

Faux Pas Task

The Faux Pas task consists of 10 short descriptions of social situations. Participants have to answer questions about the situation, for example 'Did anyone say something he/she better shouldn't have said?' and 'Wo said something he/she shouldn't have said?'. The task involves recognizing faux pas.

Wall of Faces task

The Wall of faces task (Simmons et al, 2006) assesses a complex form of social cognition. In the task, 32 faces are presented which vary in angry/happy and male/female ratio. Participants respond whether there are more angry or happy faces or more male or female faces. There are ambiguous (16 of each face type) and unambiguous (6/26) trials. The task allows for investigation of brain activation associated with social cognitive processes.

Emotional Faces paradigm

In the emotional faces paradigm based on Fisher et al. (2014) participants complete a gender discrimination task including blocks of individual neutral, fearful, happy and angry faces. Each trial consists of a stimulus and a fixation cross. Each block comprises 6 trials, including three to five faces trials and one to three null trials consisting of a fixation cross. This task allows for examination of basal threat-related brain response.

Self-Expression and Control Scale

The Self-expression and Control scale (ZECV) measures whether participants internalize or externalize feelings of anger and to what extent they can control that anger. The ZECV consists of 40 items and participants respond by rating themselves on a scale ranging from 1 'almost never' to 4 'almost always'. The ZECV has good to high psychometric properties (van Elderen, Verkes, Arkesteijn, & Komproe, 1996).

Internalized Stigma of Mental Illness

The Internalized Stigma of Mental Illness (ISMI) questionnaire is designed to measure the subjective experience of stigma, with subscales measuring alienation, stereotype endorsement, perceived discrimination, social withdrawal and stigma resistance. The questionnaire contains 29 items. The ISMI has a high internal consistency and test-retest reliability (Ritsher, Otilingham & Grajales, 2003).

Schaal interpersoonlijk gedrag

The Schaal Interpersoonlijk Gedrag (SIG; W.A. Arrindell, P.M. de Groot & J.A. Walburg, 1984) measures interpersonal behaviour by means of 50 items divided over 4 subscales: expression of negative emotions, expression of positive emotions, expression of insecurity, express yourself. Respondents fill in the level of discomfort they feel and the frequency of occurrence of social situation-items on a five-point-scale. The scale has an internal consistency of $\alpha = .80$.

Self-Esteem Rating Scale – Short Form

The original 40-item SERS was reduced to a psychometrically good 20 item version of the SERS, the SERS-SF. The 20-item Self-Esteem Rating Scale (SERS-SF) assesses self-esteem by means of a positive and negative self-esteem subscale. The two scales have excellent internal consistency ($\alpha = .91$ positive scale; $\alpha = .87$ negative scale). The test–retest reliability of both scales is high ($r = 0.90$; $r = 0.91$). The SERS total score correlated highly with both scales ($r = 0.72$ and $r = 0.79$), indicating good convergent validity (Lecomte et al. 2006).

Brief Negative Symptom Scale

The Brief Negative Symptom Scale (BNSS) is a 13-item semi-structured interview, which has been developed to assess current level of negative symptoms in treatment studies. The instrument is an addition to the Positive and Negative Syndrome Scale (PANSS) and it consists of 6 subscales: anhedonia, distress, asociality, avolition, blunted affect and alogia.

Positive and Negative Symptom Scale

The Positive and Negative Syndrome Scale (PANSS) is administered to monitor the changes in primary symptoms. The PANSS is a 30 item rating scale based on chart review and a semi-structured interview. It is widely used for assessing positive, negative and cognitive symptoms (Kay, Flszbein, & Opfer, 1987).

Manchester Short Assessment of Quality of Life

The Manchester Short Assessment of Quality of Life (MANSA; Priebe et al., 1999) is a short version of the Lancashire Quality of Life Profile (LQLP; Oliver, 1991), and has been developed to measure quality of life of psychiatric patients. The questionnaire consists of four objective items and twelve subjective items. The objective items assess victimization and accusation of crime in the past year, whether someone has a good friend and whether the participant had contact with this friend in the past week. The subjective items assess satisfaction with life as a whole, job, financial situation, leisure activities, accommodation, personal safety, sex life, people with whom the individual lives with, relationship with family, physical health, mental health and friendships. The internal consistency is sufficient (α= .74) to good (α= .81).

Trauma Screening Questionnaire

The Trauma Screening Questionnaire (TSQ) examines whether participants can be diagnosed with post-traumatic stress disorder (PTSD). The TSQ is a short screening instrument and consists of five re-experiencing and five arousal items from the DMS-IV PTSD criteria. Participants report whether or not they experienced the item twice in the past week. The sensitivity and specificity of the TSQ are high (Dekkers, Olff, & Näring, 2010).

Nationale Herstelschaal

The Nationale herstelschaal (NHS) is a Dutch scale which is developed to assess personal recovery of individuals with (severe) mental illnesses. The 26 item scale consists of two subscales: personal recovery and recovery with regard to interpersonal relations. The instrument is reliable and valid (van Weeghel et al. 2015).

Accelerometer

Accelerography is a valuable tool for motion analysis in psychiatry (Teicher 1995). It has been used in psychosis to study sleep and circadian rhythms (Haug et al., 2000; Hofstetter et al., 2005; Martin et al., 2005; Poyurovsky et al., 2000; Shamir et al., 2000; Wulff et al., 2006). However, data on quantitative motor activity can also be obtained using accelerometers (Farrow et al., 2005; Farrow et al., 2006; Walther et al., 2009). All subjects will wear an accurate and validated accelerometer for the continuous recording of motor activity. The accelerometer is a light and small device which can be worn at the wrist, ankle or hip.

Modified Shuttle Test

The Modified Shuttle Test is a sub-maximal test to measure endurance. Participants have to walk between two points. A beep tells when the participant has to be at the other point. The interval between the beeps becomes shorter every beep. The MST has been studied and is appropriate for people who suffer from somatic disorders or decreased fitness (Campo, Chilingaryan , Berg , Paradis, & Mazer, 2006; Klijn, Van der Baan-Slootweg, & Van Stel, 2007).

Simple Physical Activity Questionnaire (SIMPAQ)

The Simple Physical Activity Questionnaire contains eight questions regarding physical activity during the past seven days. The aim of including the questionnaire is to investigate its validity.

6.1.3 Main study parameters/end point MRI-part

MRI is a non-invasive way to study and visualize the structure of the living human brain. This method measures the concentration of water in different tissue types, based on the magnetic resonance of protons. Functional MRI permits the visualization of brain regions activated during the performance of a specific (mental) act, in this case during the performance of a social judgement task and an facial expression task. The method is based on a change in local blood oxygen perfusion in the activated region. The measure of interest is called blood oxygenation level dependent (BOLD) signal. Due to a poor signal to noise ratio, a single measurement is insufficient. Therefore, the brain is scanned a large number of times during an fMRI scan in order to improve the signal to noise ratio. In addition to functional scans, an (T1-weighed) anatomical scan is required in order to localize functional activation.

6.1.4 Covariates

To see what factors influence the results, demographic and biographic variables and substance use will be included in the assessment (Table 3). Alcohol and drugs use is measured before and after the intervention in case something changes during the treatment. Substance use is measured with the “Screening risico op verslavingsproblemen” (Spijkerman, Hendriks, van der Gaag, 2011) and consist of eleven questions regarding how much alcohol and drugs the participant uses in one week or month. Questions about gender, family contact, age, medication and residential area are only asked at baseline. .

Table 3: covariates

**Parameter**	**Instrument**	**Duration**	**Type**
Alcohol drugs use	Screening Risico	10 minutes	Self-rating

	Verslavingsproblemen		
-Gender<br>-Family contact<br>-Age<br>-Medication<br>-Safety residential area *	Biographical questionnaire	10 minutes	Open questions and rating scale

* based on neighbourhood crime statistics from IVM obtained from the police department.

6.2 Randomisation, blinding and treatment allocation

Participants will be allocated to either the treatment group or control group at random by means of randomisation on the factors: site, gender and participation in fMRI substudy. We will correct for randomisation factors in the analysis, in order to prevent bias. At all sites experienced interviewers are available that will be blinded to the study condition. Interviewers remain blind to the treatment allocation, but patients cannot be blinded after care allotment.

6.3 Study procedures

Chapter 2 shows a flow chart of the study design and main procedures. Onsite therapists will screen patients based on seven selection questions. Selected patients will be contacted and asked if they are interested in participating in the study. Interested patients will be sent an information letter. Two weeks after receipt of the information letter, patients will be contacted again to ask whether they decided to participate in the study and whether they would like to participate in the additional MRI sub-study. Subsequently, a selection assessment is planned in order to check whether patients meet the inclusion criteria by means of two interviews and if applicable, to fill out an MRI-checklist. When patients are eligible for participation, an informed consent form will be obtained.

If a patient does not meet the criteria, he/she will be excluded from the study. The assessor will call the patient and tell that he/she cannot participate but will receive 10 euros compensation for the selection assessment. With patients who meet the selection criteria a baseline assessment is planned. During this appointment of approximately 160 minutes, patients will fill out several questionnaires. During this appointment, participants receive an accelerometer.

They will be instructed to hand in the device at the following appointment. Patients who are also interested in participating in the MRI sub-study and meet the additional MRI criteria, are asked for a pretest MRI session. During this appointment of approximately 90 minutes participants will perform two tasks in an MRI scanner. After the pretest(s), participants will either receive the psychomotor assertiveness training consisting of 20 weekly training sessions (treatment-group) or befriending sessions (control-group). The last session, participants again receive an accelerometer. They will be instructed to hand in the device at the post assessment. Within two weeks after the last intervention session, a posttest assessment will take place. The appointment will take approximately 180 minutes. With patients who also participate in the MRI sub-study, a posttest MRI session will be planned. After six months, participants will be contacted again for a follow-up assessment consisting of questionnaires similar to the earlier pre- and posttest assessment. Respectively, a year and two years later, participants will be contacted for a second and third follow-up assessment. These assessments will take approximately 110 minutes and consists of an interview and questionnaires. More details about the selection and pre- and posttest instruments can be found in paragraph 5.1. Table 5 shows the study design of the instruments (see appendix F for instruments). The selection assessment, pre- and posttest assessments, follow-up assessments with questionnaires and the intervention sessions will take place at the mental health care institutions. The MRI sub-study will take place at the Neuroimaging Centre in Groningen.

Table 5: study design of the instruments

**Instruments**	**Pre-selection**	**Selection**	**Pre-assessment**	**Post-assessment**	**Follow-up I**	**Follow-up II**	**Follow-up III**
Selection criteria evaluated by onsite therapist	x						
Positive and negative symptoms *(PANSS)		x		x	x	x	x
Brief negative symptom scale * (BNSS)		x		x	x	x	x
MiniSCAN		x					
Integrale veiligheidsmonitor (IVM)			x	x	x	x	x
Revised Conflict Tactics Scale (CTS2)			x	x	x	x	x
Self-expression and control (ZECV)			x	x	x		
Internalized stigma of mental illness (ISMI)			x	x	x		
Self-esteem rating scale – short form (SERS-SF)			x	x	x		
Screening Risico Verslavingsproblemen (Substance abuse)			x	**x**	x	x	x

Schaal Interpersoonlijk Gedrag (SIG)			x	x	x		
Faux pas task			x	x	x		
Wall of faces task**			x	x			
Emotional faces paradigm**			x	x			
Trauma Screening Questionnaire (TSQ)			x	x	x	x	x
Social functioning scale (SFS)			x	x	x	x	x
Nationale herstelschaal (NHS)			x	x	x	x	x
Manchester short quality of life (MANSA)			x	x	x	x	x
Biographical questionnaire			x				
Accelero meter			x	x			
Other type of accelero meter		x					
Modified Shuttle Test (MST)			x	x			
Simple physical activity questionnaire (SIMPAQ)		x	x				
Montreal Cognitive Assessment (MoCA)		x					
DSM 5 vragenlijst algemene psychiatrische symptomen		x					

* *PANSS and BNSS scores obtained during selection are also used as baseline measure.*
** *In case of participation in the MRI-substudy*

6.4 Withdrawal of individual participants

Participants can leave the study at any time for any reason if they wish to do so, without any consequences. The investigator can decide to withdraw participant from the study for urgent medical reasons.

6.5 Replacement of individual participants after withdrawal

Participants who withdraw from the study, will not be replaced.. Participants who withdraw from the study will still be invited for post-treatment assessment and follow-up.

7. SAFETY REPORTING

7.1 Section 10 WMO event

In accordance to section 10, subsection 1, of the WMO, the investigator will inform the participants and the reviewing accredited METC if anything occurs, on the basis of which it appears that the disadvantages of participation may be significantly greater than was foreseen in the research proposal. The study will be suspended pending further review by the accredited METC, except insofar as suspension would jeopardise the participants' health. The investigator will take care that all participants are kept informed.

7.2 Adverse events (AEs)

None of the previous studies on exercise therapy or PMT, nor the feasibility study indicate any risk of adverse events. Furthermore, we do not foresee any problems caused by kickboxing. Although kickboxing is sometimes associated with aggression in the media there is no scientific prove that kickboxing itself induces aggressive behaviour. As shown in chapter 1 there are other explanations for the associated aggression. This is consistent with the findings of the pilot. In no way this training has been harmful for patients or for the people in their environment. Participants find the training enjoyable and experience a decrease rather than an increase in anger and aggression.

To prevent physical injuries, all exercises are carefully build-up during the training and participants can participate on their own physical level. It is possible that some participants will experience minor muscle aches after the training, especially those who are not used to physical exercise. Although participants learn how to be careful, adapt their kickboxing moves and use boxing gloves, shin guards and punching bags it is possible that someone will get a small bruise. Both the muscle ache and the bruise will disappeared in a few days and are not considered as health risks.

As shown in the exclusion criteria, in case of pregnancy, a participant cannot participate.

In case anything unforeseen happen, CCMO will be informed immediately. All adverse events reported spontaneously by the participant or observed by the investigator or his staff will be recorded.

8. STATISTICAL ANALYSIS

8.1 Descriptive statistics

Descriptive statistics will be provided for all normal distributed variables in the form of mean scores (and standard deviations) before and after the intervention on each of the assessment instruments. Descriptive statistics of variables that are not normally distributed will be represented as median scores and interquartile range will be used. Special attention will be given to numbers and characteristics of participants resigning from the study.

8.2 Analysis

The effect of the training on long term victimization (primary study parameter) and on general long term outcomes, will be investigated by means of a repeated measures fixed-effects analysis of variance (ANOVA). The effect on risk factors of victimization (second study parameter) will be tested using multilevel modelling with the pre- and posttest and follow-up on the first level and training condition on the second level (o.a. Snijders & Bosker, 2000). A model will be constructed for every dependent variable and the dependent variables will be adjusted for covariates. Statistical analysis will be conducted using the IBM SPSS Statistics package. Dummy variables will be constructed for every level and the statistical significance of the regression-effects will be tested using the T-test. The dummy-variables and their interaction will be added to the model as fixed effects. The level of significance is set at $p<0.05$, two-sided.

8.3 fMRI Analysis

The neuroimaging data will be analyzed using statistical parametric mapping 8 (SPM 8) (Wellcome Department of Cognitive Neurology London, UK; http://www.fil.ion.ucl.ac.uk). Preprocessing will include realignment, coregistration, spatial normalization into a standard space and smoothing. We will use traditional General Linear Model (GLM) voxel-wise analyses that will allow for the identification of threat-related brain activity. Baseline victimization scores as measured by the IVM (integrale veilgheidsmonitor) will be entered as regressors. Moreover, the effects of the training will be evaluated via 2 (Group: intervention, control)×2 (Time: pre-, post intervention) interaction tests. Furthermore, we will study intrinsic connectivity during a resting state fMRI scan as a measure of brain system integrity. We will use region-of-interest (ROI) analyses for the amygdala to test our a priori hypotheses. The statistical thresholds we will employ are $p < .05$ (smallvolume corrected) for ROI analyses and $p<.001$ for whole-brain analyses.

ETHICAL CONSIDERATIONS

8.4 Regulation statement

The study will be conducted according to the principles of the Declaration of Helsinki (version 10, October 19th 2013) and in accordance with the Medical Research Involving Human Subjects Act (WMO) and other guidelines, regulations and Acts (Wet Geneeskundige Behandelingsovereenkomst, Wet Bescherming Persoonsgegevens, Wet Beroepen Individuele Gezondheidszorg, Kwaliteitswet Zorginstellingen, Privacyreglement van de zorginstelling).

8.5 Recruitment and consent

Chapter 2 shows a flow chart of the study design and main procedures. First the onsite therapist will answer seven screening questions about their patients (see appendix F1). When the answers are in accordance with the selection criteria (see 3.2 and 3.3 for inclusion and exclusion criteria) the patient will receive a phone call from his/her psychomotor therapeut or another caregiver from his/her mental health institution. The assessor will inform the patients about the study and ask them if they are interested in participation and if they would like to receive an information letter about the study (see appendix E2). The assessor will send this letter by mail or email depending on what the patient prefers. Patients have two weeks to consider their participation. After two weeks, patients are contacted again to ask whether they decided to participate in the study.

During the first phone call all patients are also invited for a one hour information meeting. In this meeting patients will get more detailed information about the study procedures and the intervention and get acquainted with the trainers. The patients are invited to bring family and/or friends to this meeting in order to support and motivate patients for participation. Also, their questions about the intervention can be answered. All the involved mental health care professionals will also receive an invitation for the meeting (see appendix E3). When patients have second thoughts after the meeting they are free to refrain. After the information meeting the assessor will call all the interested patients to make an appointment for the selection assessment. The assessor will tell them again about the study procedures, randomization and intervention in case something was not clear during the meeting or someone did not attend the meeting. In addition, the patients are told that the intervention is in an experimental phase, and that no guarantees can be given regarding decreasing victimization risk. The selection assessment will start two to six weeks after the information meeting. On the day of the assessment the assessor first asks the patients to read and sign an informed consent form (see appendix E1).

8.6 Benefits and risks assessment, group relatedness

More than half of the patients with a psychotic disorder will be a victim of crime once in their live. Once being a victim the chances are high that it will happen again. Victimization can have a major impact on the lives of people with a psychotic disorder. It may worsen the psychiatric symptoms and clinical course of the patients. We expect that the psychomotor assertiveness training will prevent (re)victimization of people with a psychotic disorder and will increase social participation. Questionnaires during the assessments may be confronting or tiring. Possible risks of participation in the treatment condition may be minor muscle pain or bruises, despite the use of protection. With regard to the MRI substudy, participants may experience the noise from the scanner as unpleasant.

8.7 Compensation for injury

The UMCG and University of Groningen have a participant insurance which covers all the participants in the Beat victimization project (see appendix G1). The sponsor, as in UMCG/RUG, has a liability insurance (see appendix G2).
The sponsor/investigator has a liability insurance which is in accordance with article 7 of the WMO.

The sponsor has an insurance which is in accordance with the legal requirements in the Netherlands (Article 7 WMO and the Measure regarding Compulsory Insurance for Clinical Research in Humans of 1st July 2015). This insurance provides cover for damage to research participants through injury or death caused by the study.

1. € 650.000,-- (i.e. six hundred and fifty thousand Euro) for death or injury for each participant who participates in the Research;
2. € 5.000.000,-- (i.e. five million hundred thousand Euro) for death or injury for all participants who participate in the Research;
3. € 7.500.000,-- (i.e. seven million five hundred thousand Euro) for the total damage incurred by the organisation for all damage disclosed by scientific research for the Sponsor as 'verrichter' in the meaning of said Act in each year of insurance coverage.

The insurance applies to the damage that becomes apparent during the study or within four years after the end of the study.

8.8 Incentives

All participants in the study will receive a monetary compensation for the assessment of the pretest and posttests and follow-up. For each pre/posttest patients receive 20 euros, for the on line follow-up they receive 10 euros. Participants who participate in the fMRI-part receive 20 euros for both the pre- and posttest. This results in a total of 100 euros monetary compensation for participating in the whole study. Only patients who do not meet the selection criteria, will receive 10 euros compensation for the selection assessment.

9. ADMINISTRATIVE ASPECTS, MONITORING AND PUBLICATION

9.1 Handling and storage of data and documents

Data will be handled confidentially and complies with the Dutch Personal Data Protection Act (in Dutch: De Wet Bescherming Persoonsgegevens, WBP). The project leader will give each participant a unique identification code.

Only the initial screening forms and informed consent forms contain personal information (name, date of birth). All assessment data will be coded with the participants unique identification code and will be stored anonymously. The key to the code and a participant identification list will be safeguarded by the project manager who coordinates the data-collection. During treatment data will be stored and locked at the site where the treatment is provided. After treatment, data will be collected by the project manager and stored and locked at the University of Groningen. Raw research data will be saved for 15 years. Contact information will be destroyed after publishing results of the study. ("Gedragscode Gezondheidsonderzoek", (www.fmwv.nl)).

9.2 Amendments

All substantial amendments will be notified to the METC and to the competent authority.

Non-substantial amendments will not be notified to the accredited METC and the competent authority, but will be recorded and filed by the PI and sponsor.

9.3 Annual progress report

The investigator will submit a summary of the progress of the trial to the accredited METC once a year. Information will be provided on the date of inclusion of the first participant, numbers of participants included and numbers of participants that have completed the trial, serious adverse events/ serious adverse reactions, other problems, and amendments.

9.4 End of study report

The investigator will notify the accredited METC of the end of the study within a period of 8 weeks. The end of the study is defined as the last patient's last visit. In case the study is ended prematurely, the investigator will notify the accredited METC within 15 days, including the reasons for the premature termination. Within one year after the end of the study, the investigator/sponsor will submit a final study report with the results of the study, including any publications/abstracts of the study, to the accredited METC.

REFERENCES

Bengtsson-Tops, A., & Ehliasson, K. (2012). Victimization in individuals suffering from psychosis: A swedish cross-sectional study. *Journal of Psychiatric and Mental Health Nursing, 19*(1), 23-30. doi:10.1111/j.1365-2850.2011.01749.x

Birchwood, M., Smith, J., Cochrane, R., Wetton, S. & Copestake, S. (1990). The social functioning scale. The development and validation of a new scale of social adjustment for use in family intervention programmes with schizophrenic patients. *British journal of psychiatry, 7,* 853-859.

Brecklin, L. R. (2008). Evaluation outcomes of self-defense training for women: A review. *Aggression and Violent Behavior, 13*(1), 60-76. doi:10.1016/j.avb.2007.10.001

Brekke, J. S., Prindle, C., Bae, S. W., & Long, J. D. (2001). Risks for individuals with schizophrenia who are living in the community. *Psychiatric Services, 52*(10), 1358-1366. doi:10.1176/appi.ps.52.10.1358

Brekke, J. S., Prindle, C., Bae, S. W., & Long, J. D. (2002). Risk for individuals with schizophrenia who are living in the community. *Psychiatric Services, 53*(4) doi:10.1176/appi.ps.53.4.485

Brohan, E., Elgie, R., Sartorius, N., & Thornicroft, G. (2010). Self-stigma, empowerment and perceived discrimination among people with schizophrenia in 14 european countries: The GAMIAN-europe study. *Schizophrenia Research, 122*(1-3), 232-238. doi:10.1016/j.schres.2010.02.1065

Busschbach, J. T. v., Wiersma, D., & Slooff, C. (2012). Incidents with violence against clients in treatment for psychosis using a routine outcome monitor to assess prevalence, context and professional awareness. presentation at the XIV symposium world society of victimology, the hague, 22 may 2012.

CBS (2009) Integrale veiligheidsmonitor 2008. Landelijke rapportage. [Safety monitor 2008. National report]. Den Haag: Centraal Bureau voor de Statistiek.

Chapple, B., Chant, D., Nolan, P., Cardy, S., Whiteford, H., & McGrath, J. (2004). Correlates of victimisation amongst people with psychosis. *Social Psychiatry and Psychiatric Epidemiology, 39*(10), 836-840.

Choe, J. Y., Teplin, L. A., & Abram, K. M. (2008). Perpetration of violence, violent victimization, and severe mental illness: Balancing public health concerns. *Psychiatric Services (Washington, D.C.), 59*(2), 153-164. doi:10.1176/appi.ps.59.2.153; 10.1176/appi.ps.59.2.153

Cohen, C. I., Palekar, N., Barker, J., & Ramirez, P. M. (2012). The relationship between trauma and clinical outcome variables among older adults with schizophrenia spectrum disorders. *The American Journal of Geriatric Psychiatry, 20*(5), 408-415. doi:10.1097/JGP.0b013e318211817e

Damhuis, N., van Megen, H. J. G. M., Peeters, C. F. W., & Vollema, M. G. (2011). De MiniScan als psychiatrische interventie: Pilotonderzoek naar de toegevoegde waarde van een gecomputeriseerd classificatiesysteem. = is the MiniScan a useful psychiatric intervention? A pilot study of the incremental validity of a computerised classification system. *Tijdschrift Voor Psychiatrie, 53*(3), 175-180.

Dean, K., Moran, P., Fahy, T., Tyrer, P., Leese, M., Creed, F., . . . Walsh, E. (2007). Predictors of violent victimization amongst those with psychosis. *Acta Psychiatrica Scandinavica, 116*(5), 345-353. doi:10.1111/j.1600-0447.2007.01078.x

Dekkers, A. M. M., Olff, M., & Näring, G. W. B. (2010). Identifying persons at risk for PTSD after trauma with TSQ in the netherlands. *Community Mental Health Journal, 46*(1), 20-25.

DePrince, A. P. (2005). Social cognition and revictimization risk. *Journal of Trauma & Dissociation, 6*(1), 125-141. doi:10.1300/J229v06n01_08

Dolan, M., O'Malley, K., & McGregor, K. (2013). The role of psychopathic traits and substance abuse in predicting violent victimization in patients with schizophrenia spectrum disorders. *Personality and Mental Health, 7*(1), 28-38. doi:10.1002/pmh.1220

Eddehbia, H., & Van der Putte, R. (2009). *Vechten voor je toekomst. ACB kenniscentrum voor emancipatie en participatie*.

Egan, S. K., & Perry, D. G. (1998). Does low self-regard invite victimization? *Developmental Psychology, 34*(2), 299-309. doi:10.1037/0012-1649.34.2.299

Ekinci, O., & Ekinci, A. (2013). Association between insight, cognitive insight, positive symptoms and violence in patients with schizophrenia. *Nordic Journal of Psychiatry, 67*(2), 116-123. doi:10.3109/08039488.2012.687767

Elling, A. H. F., Wisse, E., & Berk, H. v. d. (2010). *Beloften van vechtsport: Onderzoek in het kader van het programma 'tijd voor vechtsport' in opdracht van de KNKF*. Nieuwegein: Arko Sports Media.

Endresen, I. M., & Olweus, D. (2005). Participation in power sports and antisocial involvement in preadolescent and adolescent boys. *Journal of Child Psychology and Psychiatry, 46*(5), 468-478. doi:10.1111/j.1469-7610.2005.00414.x

Evangeli, M., & Broks, P. (2000). Face processing in schizophrenia: Parallels with the effects of amygdala damage. *Cognitive Neuropsychiatry, 5*(2), 81-104. doi:10.1080/135468000395754

Falconer, E., Bryant, R., Felmingham, K. L., Kemp, A. H., Gordon, E., Peduto, A., . . . Williams, L. M. (2008). The neural networks of inhibitory control in posttraumatic stress disorder. *Journal of Psychiatry & Neuroscience, 33*(5), 413-422.

Fisher, P.M., Madsen, M.K., Mahon, B., Holst, K.K., Anderson, S.B., Laursen, H.R., Hasholt, L.F., Siebner, H.R., Knudsen, G.M. (2014). Three-week bright-light intervention has dose-related effects on threat-related corticolimbic reactivity and functional coupling. *Biological Psychiatry, 76*, 332-339.

Fitzgerald, P. B., de Castella, A. R., Filia, K. M., Filia, S. L., Benitez, J., & Kulkarni, J. (2005). Victimization of patients with schizophrenia and related disorders. *Australian and New Zealand Journal of Psychiatry, 39*(3), 169-174. doi:10.1111/j.1440-1614.2005.01539.x

Hiday, V. A., Swartz, M. S., Swanson, J. W., Borum, R., & Wagner, H. R. (1999). Criminal victimization of persons with severe mental illness. *Psychiatric Services (Washington, D.C.), 50*(1), 62-68.

Hoekert, M., Kahn, R. S., Pijnenborg, M., & Aleman, A. (2007). Impaired recognition and expression of emotional prosody in schizophrenia: Review and meta-analysis. *Schizophrenia Research, 96*(1-3), 135-145. doi:10.1016/j.schres.2007.07.023

Holley, J., Crone, D., Tyson, P., & Lovell, G. (2011). The effects of physical activity on psychological well-being for those with schizophrenia: A systematic review. *British Journal of Clinical Psychology, 50*(1), 84-105. doi:10.1348/014466510X496220

Honkonen, T., Henriksson, M., Koivisto, A., Stengård, E., & Salokangas, R. K. R. (2004). Violent victimization in schizophrenia. *Social Psychiatry and Psychiatric Epidemiology, 39*(8), 606-612. doi:10.1007/s00127-004-0805-x

Hornsveld, R. H. J., Muris, P., & Kraaimaat, F. W. (2011). The novaco anger Scale–Provocation inventory (1994 version) in dutch forensic psychiatric patients. *Psychological Assessment, 23*(4), 937-944.

Kamperman, A. M., Henrichs, J., Bogaerts, S., Lesaffre, E. M., Wierdsma, A. I., Ghauharali, R. R., . . . Mulder, C. L. (2014). Criminal victimisation in people with severe mental illness: A multi-site prevalence and incidence survey in the netherlands. *PloS One, 9*(3), e91029. doi:10.1371/journal.pone.0091029; 10.1371/journal.pone.0091029

Kay, S. R., Flszbein, A., & Opfer, L. A. (1987). The positive and negative syndrome scale (PANSS) for schizophrenia. *Schizophrenia Bulletin, 13*(2), 261-276.

Kleim, B., Vauth, R., Adam, G., Stieglitz, R., Hayward, P., & Corrigan, P. (2008). Perceived stigma predicts low self-efficacy and poor coping in schizophrenia. *Journal of Mental Health, 17*(5), 482-491. doi:10.1080/09638230701506283

Lakes, K. D., & Hoyt, W. T. (2004). Promoting self-regulation through school-based martial arts training. *Journal of Applied Developmental Psychology, 25*(3), 283-302. doi:10.1016/j.appdev.2004.04.002

Lamarre, B. W., & Nosanchuk, T. A. (1999). Judo—the gentle way: A replication of studies on martial arts and aggression. *Perceptual and Motor Skills, 88*(3), 992-996. doi:10.2466/PMS.88.3.992-996

Lecomte T, Corbiere M, Laisne F: Investigating self-esteem in individuals with schizophrenia: relevance of the Self-Esteem Rating Scale-Short Form. Psychiatry Res 2006, 143:99–108.

Livingston, J. D., & Boyd, J. E. (2010). Correlates and consequences of internalized stigma for people living with mental illness: A systematic review and meta-analysis. *Social Science & Medicine, 71*(12), 2150-2161. doi:10.1016/j.socscimed.2010.09.030

Makin-Byrd, K., Bonn-Miller, M., Drescher, K., & Timko, C. (2012). Posttraumatic stress disorder symptom severity predicts aggression after treatment. *Journal of Anxiety Disorders, 26*(2), 337-342. doi:10.1016/j.janxdis.2011.11.012

Malchow, B., Reich-Erkelenz, D., Oertel-Knöchel, V., Keller, K., Hasan, A., Schmitt, A., . . . Falkai, P. (2013). The effects of physical exercise in schizophrenia and affective disorders. *European Archives of Psychiatry and Clinical Neuroscience, 263*(6), 451-467. doi:10.1007/s00406-013-0423-2

Marzolini, S., Jensen, B., & Melville, P. (2009). Feasibility and effects of a group-based resistance and aerobic exercise program for individuals with severe schizophrenia: A multidisciplinary approach. *Mental Health and Physical Activity, 2*(1), 29-36. doi:10.1016/j.mhpa.2008.11.001

Monger, B., Hardie, S.M., Ion, R., Cumming, J. & Henderson, N. (2013). The individual recovery outcomes counter: preliminary validation of a personal recovery measure. *The Psychiatrist, 31,* 221-227.

Monti, M.M. Statistical analysis of fMRI time-series: A critical review of the GLM approach. Front Human Neuroscience. 2011, 5:28.

Nosanchuk, T. A., & MacNeil, M. C. (1989). Examination of the effects of traditional and modern martial arts training on aggressiveness. *Aggressive Behavior, 15*(2), 153-159. doi:10.1002/1098-2337(1989)15:2<153::AID-AB2480150203>3.0.CO;2-V

Perala, J., Suvisaari, J., Saarni, S. I., Kuoppasalmi, K., Isometsa, E., Pirkola, S., . . . Lönnqvist, J. (2007). *Lifetime prevalence of psychotic and bipolar I disorders in a general population*. US: American Medical Assn.

Peri, T., Ben-Shakhar, G., Orr, S. P., & Shalev, A. Y. (2000). Psychophysiologic assessment of aversive conditioning in posttraumatic stress disorder. *Biological Psychiatry, 47*(6), 512-519. doi:10.1016/S0006-3223(99)00144-4

Phillips, L. K., & Seidman, L. J. (2008). Emotion processing in persons at risk for schizophrenia. *Schizophrenia Bulletin, 34*(5), 888-903. doi:10.1093/schbul/sbn085

Ritsher, J.B., Otilingham, P.G. & Grajales, M. (2003). Internalized stigma of mental illness: psychometric properties of a new instrument. *Psychiatry Research, 121*, 31-49.

Röhricht, F. (2009). Body oriented psychotherapy. the state of the art in empirical research and evidence-based practice: A clinical perspective. *Body, Movement and Dance in Psychotherapy, 4*(2), 135-156. doi:10.1080/17432970902857263

Röhricht, F., Papadopoulos, N., Suzuki, I., & Priebe, S. (2009). Ego-pathology, body experience, and body psychotherapy in chronic schizophrenia. *Psychology and Psychotherapy: Theory, Research and Practice, 82*(1), 19-30. doi:10.1348/147608308X342932

Scheewe, T. W., Backx, F. J. G., Takken, T., Jörg, F., van Strater, A. C. P., Kroes, A. G., . . . Cahn, W. (2013). Exercise therapy improves mental and physical health in schizophrenia: A randomised controlled trial. *Acta Psychiatrica Scandinavica, 127*(6), 464-473. doi:10.1111/acps.12029

Short, T. B. R., Thomas, S., Luebbers, S., Mullen, P., & Ogloff, J. R. P. (2013). A case-linkage study of crime victimisation in schizophrenia-spectrum disorders over a period of deinstitutionalisation. *BMC Psychiatry, 13*

Simmons, A., Stein, M.B., Matthews, S.C., Feinstein, J.S. & Paulus, M.P. (2006). Affective ambiguity for a group recruits ventromedial prefrontal cortex. *Neuroimage, 29*, 655 – 661.

Straus, M. A., Hamby, S. L., Boney-McCoy, S., & Sugarman, D. B. (1996). The revised conflict tactics scales (CTS2): Development and preliminary psychometric data. *Journal of Family Issues, 17*(3), 283-316.

Twemlow, S. W., Biggs, B. K., Nelson, T. D., Vernberg, E. M., Fonagy, P., & Twemlow, S. W. (2008). Effects of participation in a martial arts-based antibullying program in elementary schools. *Psychology in the Schools, 45*(10), 947-959. doi:10.1002/pits.20344

Thirion, B., Pinel, P., Mériaux, S., Roche, A., Dehaene, S. & Poline, J.B.I. (2007). Analysis of a large fMRI cohort: Statistical and methodological issues for group analyses. *Neuroimage, 35,* 105-120.

van Dam-Baggen, R., & Kraaimaat, F. (1999). Assessing social anxiety: The inventory of interpersonal situations (IIS). *European Journal of Psychological Assessment, 15*(1), 25-38.

van Elderen, T., Verkes, R. J., Arkesteijn, J., & Komproe, I. (1996). Psychometric characteristics of the self-expression and control scale in a sample of recurrent suicide attempters. *Personality and Individual Differences, 21*(4), 489-496.

Vega, E. M., & O'Leary, K. D. (2007). Test-retest reliability of the revised conflict tactics scales (CTS2). *Journal of Family Violence, 22*(8), 703-708.

Vertonghen, J., & Theeboom, M. (2010). The social-psychological outcomes of martial arts practise among youth: A review. *Journal of Sports Science and Medicine, 9*(4), 528-537.

Vertonghen, J., & Theeboom, M. (2012). Martial arts and youth: An analysis of contextual factors. *International Journal of Adolescence and Youth, 17*(4), 237-241. doi:10.1080/02673843.2012.687689

Volavka, J., & Citrome, L. (2011). Pathways to aggression in schizophrenia affect results of treatment. *Schizophrenia Bulletin, 37*, 921-929. doi:10.1093/schbul/sbr041

Walsh, E., Moran, P., Scott, C., McKenzie, K., Burns, T., Creed, F., . . . Fahy, T. (2003). Prevalence of violent victimisation in severe mental illness. *The British Journal of Psychiatry, 183*(3), 233-238. doi:10.1192/bjp.183.3.233

Weeghel, J. v., Kamperman, A., Vries, S. d., Plooy, A., & Mulder, C. (2009). *Vooronderzoek geweld tegen psychiatrische patiënten. NWO onderzoeksprogramma*. Rotterdam/Utrecht: NWO:

Witt, K., van Dorn, R., & Fazel, S. (2013). Risk factors for violence in psychosis: Systematic review and meta-regression analysis of 110 studies. *PLoS ONE, 8*(2)

Zivin, G., Hassan, N. R., DePaula, G. F., Monti, D. A., Harlan, C., Hossain, K. D., & Patterson, K. (2001). An effective approach to violence prevention: Traditional martial arts in middle school. *Adolescence, 36*(143), 443-459.
